# Federated learning with differential privacy for breast cancer diagnosis enabling secure data sharing and model integrity

**DOI:** 10.1038/s41598-025-95858-2

**Published:** 2025-04-16

**Authors:** Shubhi Shukla, Suraksha Rajkumar, Aditi Sinha, Mohamed Esha, Konguvel Elango, Vidhya Sampath

**Affiliations:** 1 School of Electrical Engineering, Vellore Institute of Technology, Vellore, 632014 India; 2 School of Electronics Engineering, Vellore Institute of Technology, Vellore, 632014 India; 3 School of Mechanical Engineering, Vellore Institute of Technology, Chennai, 600127 India

**Keywords:** Federated learning, Data security, Privacy preservation, Healthcare, Decentralized machine learning, Differential privacy, Breast cancer, Health care, Diagnosis, Computational science, Computer science, Scientific data, Statistics

## Abstract

In the digital age, privacy preservation is of paramount importance while processing health-related sensitive information. This paper explores the integration of Federated Learning (FL) and Differential Privacy (DP) for breast cancer detection, leveraging FL’s decentralized architecture to enable collaborative model training across healthcare organizations without exposing raw patient data. To enhance privacy, DP injects statistical noise into the updates made by the model. This mitigates adversarial attacks and prevents data leakage. The proposed work uses the Breast Cancer Wisconsin Diagnostic dataset to address critical challenges such as data heterogeneity, privacy-accuracy trade-offs, and computational overhead. From the experimental results, FL combined with DP achieves 96.1% accuracy with a privacy budget of ε = 1.9, ensuring strong privacy preservation with minimal performance trade-offs. In comparison, the traditional non-FL model achieved 96.0% accuracy, but at the cost of requiring centralized data storage, which poses significant privacy risks. These findings validate the feasibility of privacy-preserving artificial intelligence models in real-world clinical applications, effectively balancing data protection with reliable medical predictions.

## Introduction

Artificial Intelligence (AI) has revolutionized numerous industries, with the healthcare industry being the most promising sector for AI implementation. AI models have proven to be highly efficient in improving diagnostic accuracy, optimizing treatment plans, and improving medical research. However, traditional AI models require centralized data storage, where patient information is aggregated in a single repository. This centralized approach raises concerns regarding patient privacy, security risks, and regulatory compliance, especially in the healthcare sector, where sensitive medical data is involved. With the growing emphasis on data security, there is a requirement for decentralized learning approaches that can support collaborative AI training while ensuring data confidentiality.

Federated Learning (FL) and Differential Privacy (DP) are two of such approaches that address these challenges by enabling the development of AI that preserves privacy in healthcare. FL allows multiple medical institutions to collaboratively train a shared AI model without transferring raw patient data to a central server, thereby mitigating the risk of data breaches^1,2^. Instead, model updates are exchanged and aggregated, retaining patient data in a local hospital environment. Think of Differential Privacy as the process of adding a slight blur to a picture, just enough to obscure distinctive details but preserve the overall pattern. DP achieves this by adding statistical noise to model updates, preventing the identification of individual patient data^3,4^. By combining FL and DP, it is possible to develop robust AI models that respect patient privacy while maintaining predictive accuracy, making them well-suited for regulatory-compliant healthcare applications.

The increasing digitization of healthcare has led to a massive accumulation of patient-related data, including electronic health records (EHRs), diagnostic imaging, genomic data, and real-time monitoring from wearable devices^5,6^. This data-driven transformation has paved the way for predictive models, personalized treatment plans, and data-driven decision-making. However, with these advancements come significant challenges related to data privacy and security^3,4^. Stringent regulations such as the Health Insurance Portability and Accountability Act (HIPAA) in the United States and the General Data Protection Regulation (GDPR) in the European Union impose strict constraints on the sharing of medical data^6^. Traditional centralized AI models often violate these regulations because they require data transfer across institutions, increasing privacy risks. In contrast, FL-DP provides a legally compliant alternative that allows institutions to develop AI models collaboratively without violating data protection laws.

Federated Learning has emerged as a preferred solution for attaining collaborative AI training while upholding stringent privacy requirements^7,8^. In contrast to standard centralized machine learning models that rely on aggregating raw data, FL avoids local dataset sharing by any institution while only encrypted updates of the model are shared with a central aggregator^1,2^. FL not only increases security, but also avoids the risk of large-scale data breaches^5^. FL also addresses core challenges in healthcare AI deployment, such as data heterogeneity, security loopholes, and high communication overhead^9,10^. Nonetheless, even though promising, FL alone does not guarantee complete protection of data. There is a risk of model inversion attacks as well as other adversarial attacks, and thus, other privacy-maintaining measures like DP must be combined.

The primary contribution of this study is to introduce a privacy-preserving FL framework for breast cancer detection that incorporates DP to ensure secure decentralized AI training. The key advancements of our work include:

- This study introduces privacy-preserving federated learning (FL) for breast cancer detection, enabling decentralized learning without sharing sensitive patient data, ensuring secure and collaborative model development across healthcare institutions.
- The integration of Differential Privacy (DP) within the FL framework, which enhances data security while maintaining high model accuracy, effectively balances the critical trade-off between privacy and performance.
- The performance evaluation of the proposed model demonstrated superior accuracy, outperforming traditional centralized models while preserving patient privacy, which validates the effectiveness of our approach.
- The paper addresses key challenges inherent in FL, such as data heterogeneity, security risks, and communication overhead, by implementing solutions that improve model robustness and efficiency.
- A comprehensive comparison of various privacy-preserving techniques identifies DP as the most practical and effective solution for FL in healthcare applications, highlighting its superiority in maintaining both security and model performance.
- The paper proposes future directions for enhancing the FL framework, including the integration of blockchain technology for improved security, adaptive privacy tuning to optimize performance dynamically, and scalability improvements for broader deployment across diverse healthcare systems.

Federated Learning, as promising as it is, is plagued by several challenges in the heterogeneity of healthcare data. Various medical centers tend to possess datasets with non-uniform statistical patterns, a problem referred to as non-independent and identically distributed data. This lack of uniformity results in model learning disproportionately, and, as such, there will be delayed increases in accuracy^4^. Such heterogeneity complicates optimal model convergence, necessitating specialized aggregation methods and adaptive FL approaches^11–13^. Moreover, the implementation of uniform security measures in a decentralized FL network is challenging because institutions may use varying degrees of data privacy and protection^10,14,15^. Even if privacy-preserving technologies such as DP and MPC are used, over-adding noise would compromise model accuracy, necessitating the creation of an optimal trade-off between privacy protection and performance^16^.

This study confirms that FL-DP successfully preserves privacy while maintaining strong diagnostic accuracy for breast cancer detection. Our experiments indicate that while DP introduces a small accuracy trade-off, it provides robust privacy guarantees, making it an effective solution for privacy-sensitive applications. The results further demonstrate that the use of an optimal privacy budget minimizes performance loss while ensuring compliance with regulatory frameworks. FL has also been shown to outperform centralized AI models by reducing false positives and false negatives, which is crucial for clinical decision-making. Moreover, DP is computationally efficient and scalable, outperforming alternative techniques such as Homomorphic Encryption (HE) and Secure Multi-Party Computation (SMPC) in terms of practical deployment.

However, deploying FL-DP in real-world hospital environments requires overcoming key challenges, including inter-institutional collaboration barriers, standardization of FL protocols, and ensuring patient consent for federated model training^7^. Healthcare institutions vary in regulatory and technical infrastructure and applying a common FL framework across institutions is difficult. In addition, integrating FL into existing electronic health record (EHR) systems requires significant modifications in the hospital’s IT infrastructure and computational resources. Despite all this, industry advancements, such as dermatology AI trials with Google’s FL-based technology, indicate that FL can be successfully integrated into real-world clinical workflows. Future research needs to explore the integration of blockchain technology for additional security, adaptive DP mechanisms for privacy optimization, and large-scale medical dataset evaluations to facilitate mass adoption of FL-DP in healthcare^5^. By addressing these core areas, this work makes significant contributions to the building of privacy-preserving AI in healthcare towards safe, collaborative, and regulation-friendly model development for life-critical medical use cases.

## Related work

The literature survey section focuses on the papers dealing with the usage of widespread adoption of FL in healthcare applications, ensuring privacy-preserving machine learning while maintaining data security and efficiency. The problem of leakage of privacy from model updates was first identified in early research^27^, which necessitated the development of privacy-preserving techniques. The use of Federated Learning (FL) in healthcare has attracted considerable attention as it can support privacy-preserving machine learning while maintaining data security and computational efficiency. The first privacy leakage issues in FL models were identified when it was shown that adversaries could recover sensitive information from shared model updates. Various works have investigated the challenge of FL in healthcare usage, explicitly considering the privacy risk, security attack (e.g., data poisoning, model inversion), large communication cost, non-IID data, privacy-accuracy trade-offs, scalability, and compliance with regulatory requirements^17–22^. Resolving such challenges is critical to unlock the complete potential of FL in real-world medical deployments.

FL has been extensively viewed as a privacy-preserving solution to centralized machine learning in the healthcare sector, enabling institutions to jointly train models without exchanging raw patient data^23,24^. Decentralization, however, brings with it new threats, such as privacy-model accuracy trade-offs and security breaches^25,26^. Various privacy-preserving mechanisms, including Differential Privacy (DP), Homomorphic Encryption (HE), and Secure Multi-Party Computation (SMPC), have been proposed to mitigate these threats^27^. Of these, DP is extensively employed to protect privacy by adding controlled noise to model updates to avoid adversarial attacks that can reconstruct sensitive patient information. ^28^ sheds light on the need for DP to secure the medical AI applications. The research established that though DP maintains the privacy of patients, the injection of noise degrades the accuracy of the model, which calls for optimal privacy-utility trade-offs. However, over-adding noise greatly degrades model accuracy, thus creating a fundamental privacy-utility trade-off. Some research has investigated how the optimization of DP’s privacy budgets can strike a balance between privacy protection and model performance^20^.

To overcome DP’s drawbacks, cryptographic techniques like HE and SMPC have been suggested. HE supports computations on ciphertext, whereas SMPC facilitates secure collaborative training without revealing individual contributions^29^. Both methods, however, incur high computational costs, which makes them less practical for real-time medical AI applications^30^. ^31^ examine HE and SMPC in FL, noting that both techniques guarantee high privacy but incur very high computational costs, thus impractical for real-time applications, especially in medicine. Although FL supports the private processing of data, it is still vulnerable to data poisoning, model inversion attacks, and adversarial manipulations. These concerns are significant for healthcare and AI, because true, accurate diagnosis of the patients critically relies on the integrity of the data.

To further strengthen FL’s privacy and scalability, researchers have proposed blockchain-aided FL aggregation methods to defend against adversarial attacks like data poisoning and model inversion. Blockchain protects model updates from tampering but introduces extra computational delay, which restricts large-scale deployment^32^. Other research has proposed adaptive optimization methods, including bio-inspired AI methods, which adaptively adjust DP parameters to suppress accuracy deterioration while enforcing robust privacy protection^33^.

One of the latest developments in FL deployment is its integration with edge computing platforms. Cloud-based FL architectures traditionally suffer from high latency and network reliance, which slows down real-time healthcare applications. Edge computing allows FL to conduct computations near data sources (e.g., hospital imaging equipment, wearable sensors), lowering communication overhead and enhancing efficiency^34^. This method has shown improved scalability and real-time processing, making FL more feasible for clinical environments.

Despite these advancements, several challenges remain for real-world FL deployment in healthcare, including issues related to heterogeneous data distributions, inter-institutional standardization, and regulatory compliance. Several ongoing studies are exploring the use of FL-DP in radiology, pathology, and genomic research, demonstrating its scalability across various medical fields. Early findings indicate federated learning is poised to increase model generalizability across heterogeneous hospital datasets without compromising strong privacy guarantees. Such findings attest to the viability of FL-DP as a long-term, privacy-focused solution to AI-assisted diagnosis.

This work seeks to enable secure collaborative AI-based breast cancer diagnosis without revealing raw patient data, possibly preserving privacy while maintaining diagnostic accuracy. With FL-DP, healthcare organizations can securely cooperate collaboratively while meeting stringent regulatory requirements.

## Federated learning

### Overview of federated learning in healthcare

Federated learning is a machine learning approach where the data to be trained remains decentralized across multiple servers while keeping the data localized^2,34^. The clients or the servers train the model and only the updates are sent, instead of sending the raw data. Each client possesses the data locally and updates the model with the centralized server^4,17^. The central server then aggregates these updates and makes a much more efficient ML model^3,35^. This approach not only enhances privacy by keeping sensitive information within the confines of local devices but also addresses the issues inherent in traditional centralized models, such as the risk of data breaches and the challenges posed by data regulations^36^. FL also integrates the power of many decentralized devices to train the ML models, which allows for large-scale collaborations without the need for centralized data storage. The training process in FL is iterative, which means that each client trains its data locally and then sends the updated model to the server, the server then integrates it and redistributes it to the clients for further training^37^. To increase security we have used differential privacy, which adds noise to the model updates and prevents leakage of data^21^. FL is highly scalable and therefore can be applied to millions of devices, particularly useful where data is widely distributed^37–39^.

### Federated learning architecture

**Fig. 1:**
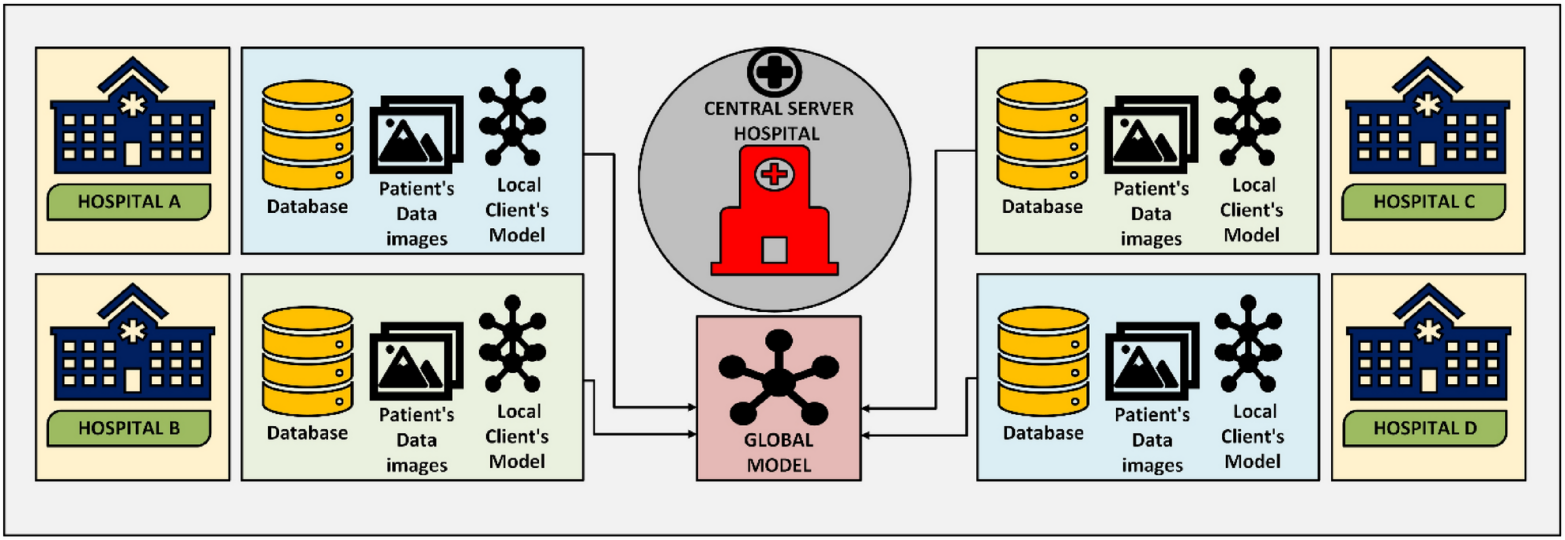
Federated learning architecture: integration of local client models with a central server to form a global model.

The architecture of Federated Learning (FL) is depicted in Fig. 1, illustrating the interaction between multiple client devices and a central server to facilitate decentralized machine learning while preserving data privacy. This architecture is particularly relevant in sensitive domains such as healthcare, where safeguarding patient data is of paramount importance^36^. The system consists of two primary components: Client Devices and the Central Server, each playing a distinct role in the federated learning process^17,40^.

Client devices are the building blocks of the federated learning system. In the decentralized system, each client—isolated in the following figure as individual hospitals (Hospital A, B, C, and D)—possesses a local dataset specific to its environment. This architecture is designed to improve data privacy by ensuring that sensitive information, such as patient records, are stored locally and never sent to remote servers^41,42^. The major tasks of client devices are storing raw data, local model training, updating the model and sending it to the central server. The process starts with each client receiving a global model from the central server^43,44^. The model is locally trained using the client’s dataset, enabling the system to learn from a heterogeneous collection of datasets without compromising privacy^45^. Once the local training process is completed, clients send only model updates, e.g., gradients and weights, to the central server for aggregation. This strategy ensures that sensitive information is secure in each client’s database, significantly minimizing the risk of data breaches^19,46^.

Even with these benefits, client devices are confronted with numerous challenges that can affect the effectiveness of the federated learning process^47,48^. One of the main challenges is the existence of non-IID (non-independent and identically distributed) data, as the datasets gathered by different clients can be highly diverse based on the data distribution and quality^49^. Such heterogeneity in data can degrade the performance and generalization ability of the global model, necessitating the use of advanced aggregation techniques to ensure robustness^50^. Furthermore, variations in computing capabilities, storage capacity, and available energy in client devices can result in skewed training times and model updates, further complicating the learning process^44,51^.

The central server is the coordinator of the federated learning system, managing communication with client devices while managing the global model. Its primary role is aggregation of model updates from the clients, followed by redistribution of the updated global model to the clients. The server does not receive raw data; instead, it performs secure aggregation of model parameters, thereby maintaining data confidentiality^52^. After receiving the model updates from all the participating clients, the central server executes aggregation algorithms, such as Federated Averaging (FedAvg), to produce an updated global model that captures the combined knowledge gained from all the clients. This updated model is then redistributed to the clients for the next round of local training, creating an iterative process that continues until the model reaches a certain level of accuracy or convergence^53^.

**Fig. 2:**
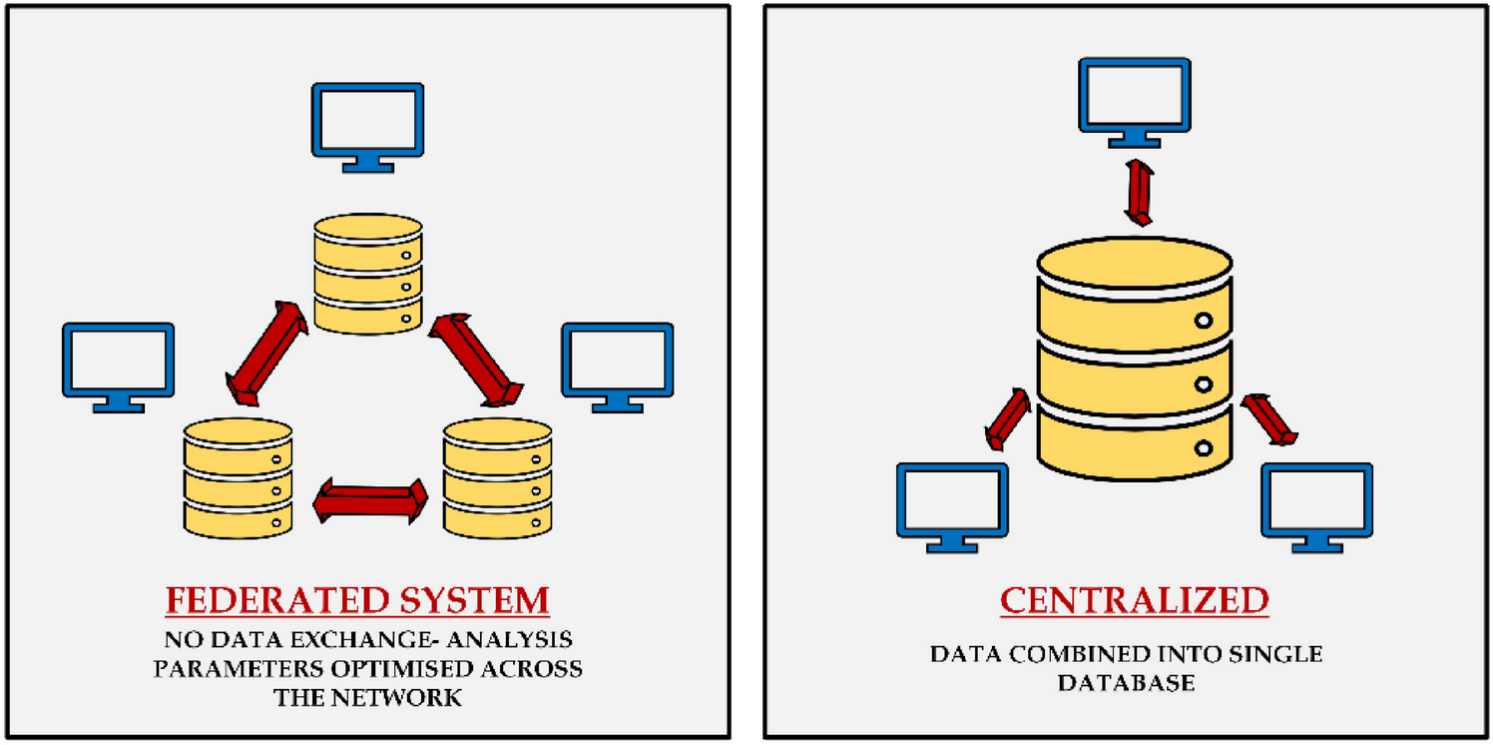
Comparative overview of federated learning and centralized learning architectures.

The role of the central server is not only important for model performance but also for system robustness and security. It is responsible for managing issues such as client dropouts, where some clients fail to send updates due to connectivity issues or hardware limitations. The server also needs to manage synchronization of model updates, ensuring the learning process is stable despite variations in client availability. Additionally, the central server plays a key role in privacy preservation, using techniques such as differential privacy and secure multi-party computation to protect sensitive data during model aggregation^54^. Figure 2 provides a comparative overview of Federated Learning (FL) and Centralized Learning (CL) architectures, highlighting the essential differences in data management, communication protocols, and privacy controls. The Federated System, illustrated on the left, is defined by data distribution across several client devices or servers^52^. Raw data is not shared directly between clients or to a server in this system^48^. Local model training is done using each client’s data set, and model updates in the form of gradients and parameters are communicated to the central server. The updates are combined to create a global model. This approach greatly enhances data privacy and security since sensitive information is not shared on the network. It also reduces bandwidth consumption since only model parameters are shared instead of large data sets. Federated learning, however, has limitations, such as communication latency, complex synchronization processes between devices, and complexity caused by non-IID (non-independent and identically distributed) data, which can affect model performance^16^.

The right side of Fig. 2 illustrates the Centralized System, where data from all devices is aggregated into a single central database. In this configuration, raw data is streamed continuously from individual devices to a master server, upon which the machine learning model is trained on the aggregated data^42,43^. Centralization is simpler to train and track models because it has access to the entire dataset, which is more likely to yield improved performance and accuracy. Centralization also circumvents the requirement for advanced synchronization mechanisms that are required in federated learning. Centralization, however, poses extreme privacy concerns because it presents one single point of compromise that can be attacked and is susceptible to data breaches. It also requires high bandwidth on data transfer and might have challenges complying with data protection regulations such as GDPR and HIPAA, particularly in sensitive sectors such as healthcare^13^.

The diagram effectively compares the two systems: the Federated System prioritizes data privacy, security, and decentralization, making it ideal for sensitive industries such as healthcare and finance. In contrast, the Centralized System focuses on performance and simplicity, making it well-suited for applications where data privacy concerns are less critical, such as social media or e-commerce analytics. Ultimately, the choice between federated and centralized learning depends on the specific application requirements, particularly the balance between data privacy and model performance.

Furthermore, Table 1 presents a detailed comparison of the key aspects differentiating federated and centralized systems, including data storage, privacy, communication requirements, and common use cases. This table provides a concise summary of the trade-offs involved in choosing between the two architectures.

**Table 1:** Comparison between federated system and centralized system.

Aspect	Federated system	Centralized system
Data Storage	Local devices (distributed)	Centralized database
Data Exchange	No raw data exchange, only model updates	Raw data is continuously sent to the central server
Privacy	High (data remains on device)	Low (data stored centrally, risk of breaches)
Communication	Optimized for model updates	High bandwidth needed for data transfer
Performance	Slightly lower due to decentralized data	High, as the model accesses the complete dataset
Use Cases	Healthcare, finance, IoT devices	E-commerce, social media, large-scale data analytics

### Challenges in federated learning

Despite its numerous advantages, Federated Learning (FL) faces several critical challenges that can impact its efficiency, security, and model performance^55^. Figure 1 illustrates how data remains decentralized, but this design introduces complexities in data distribution, communication, and security. The major challenges of FL are *data heterogeneity*, *communication overhead*, and *security issues*, which are discussed in detail in the following sections:

- *Data Heterogeneity*: One of the biggest challenges in FL is data heterogeneity, which is due to the non-independent and identically distributed (non-IID) nature of client data. In real-world FL applications, client devices (e.g., hospitals, smartphones, or IoT devices) sample data from different environments, leading to imbalanced data distributions^49,55^. Such heterogeneity can result in the deterioration of performance in the global model, as it can be biased towards datasets of clients with larger or more prominent data collections^56^. Moreover, the goal of FL is to learn a global model that demonstrates strong performance on all clients; however, this goal can be conflicting with the need for personalized models that are optimized for specific client datasets^13^. Mitigating data heterogeneity requires advanced techniques, such as personalized federated learning, client clustering, and adaptive aggregation techniques to make the global model generalize well and accommodate local data patterns.
- *Communication Overhead*: Federated Learning (FL) inherently involves periodic communication between the client devices and the central server with considerable communication overhead^57^. In contrast to traditional machine learning practices where data is centralized, FL necessitates the transmission of model updates (gradients and weights) following each training iteration. The communication process is heavily bandwidth-constrained, particularly when working with large models or a large client population^58^. Moreover, in settings where there is excessive network latency or unreliable networks, this communication acts as a bottleneck, considerably slowing model training and prolonging convergence times^59^. Model compression, update scarification, asynchronous communication, and federated dropout can alleviate communication costs; however, these practices incur costs to model performance and stability.
- *Security Concerns*: While FL vows greater data privacy through keeping raw data on client devices, FL is not immune to various security threats. In spite of employing privacy-preserving methods such as differential privacy and secure multi-party computation, FL systems remain vulnerable to malicious attacks^58,60^. For example, model poisoning attacks can arise when malicious clients deliberately send inappropriate or manipulated model updates to the central server, effectively compromising the performance of the global model or causing it to fail entirely^13,61^. Moreover, inference attacks may enable adversaries to reconstruct sensitive data from the broadcast model updates. To counteract such vulnerabilities, incorporating strong defense mechanisms such as anomaly detection, robust aggregation algorithms (e.g., Krum, Trimmed Mean), and client verification protocols is essential. However, the addition of these security practices may impose some computational overhead and complexity on the system^62^.

## Privacy preservation techniques

**Fig. 3:**
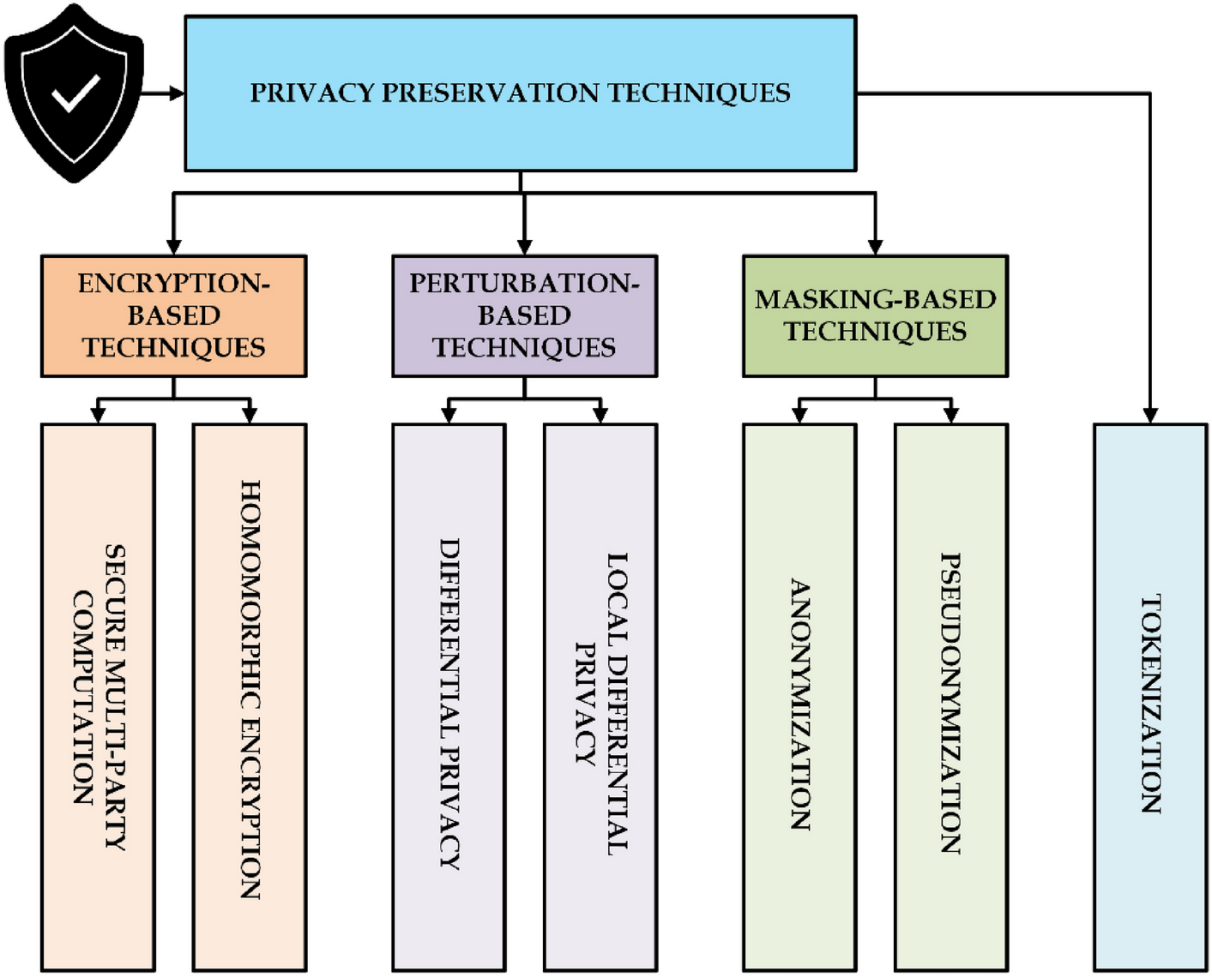
Privacy preservation techniques in federated learning.

Privacy preservation in FL is essential because the data distribution is decentralized, with many clients training models on their own and uploading only model updates rather than raw data^63^. However, in practice, privacy risks remain; malicious participants can infer sensitive information from the model gradients shared by clients or even central aggregators can compromise privacy^64^. Various techniques for preserving privacy have been developed and can be classified into three types: encryption-based, perturbation-based, and masking-based methods^27,39^. Figure 3 illustrates various privacy preservation techniques categorized into encryption-based, perturbation-based, masking-based methods, and tokenization.

### Encryption-based techniques

Encryption-based methods offer strong cryptographic guarantees. Client updates are kept confidential even when communicated and aggregated^65,66^.

- *Secure Multi-Party Computation (SMPC)*: SMPC is a technique which allows multiple parties to jointly compute a function over their inputs while keeping their data private^67^. In FL, SMPC ensures that no single entity, including the central server, gains access to individual model updates^68^. Each client encrypts its updates before sharing them, and aggregation is performed on encrypted values. However, SMPC introduces computational overhead, making it more suitable for small-scale FL applications^69^.
- *Homomorphic Encryption (HE)*: Homomorphic Encryption (HE) is a cryptographic technique that allows computations to be performed directly on encrypted data without decryption. In the context of Federated Learning (FL), HE maintains model updates encrypted even as the server aggregates them, without ever exposing raw data. Clients encrypt the model updates and transmit them, and the server aggregates the encrypted values. Although HE provides strong privacy protection by preventing the risk of data exposure, it also has significant computational overhead, which makes it more suitable in situations where data security is more important at the cost of increased processing complexity^26,64^.

### Perturbation-based techniques

Perturbation techniques introduce controlled noise into data or model updates, preventing adversaries from reconstructing sensitive information while still enabling meaningful model training.

- *Differential Privacy (DP)*: DP is widely used in FL to prevent information leakage from model updates. The Gaussian Mechanism is commonly employed, where random noise drawn from a Gaussian distribution is added to gradients before sharing them with the central aggregator. The privacy budget, represented as (*ε*, *δ*), controls the trade-off between privacy and model accuracy^70,71^. Lower (*ε*) guarantees stronger privacy in that it becomes harder to infer individual contributions, but excessive noise can degrade model performance^72^.
- *Local Differential Privacy (LDP)*: LDP is quite different from the way DP is typically applied at the central server level. In this, noise is applied directly at the client’s side before sending the updates for aggregation. So even if the update of one client is intercepted, individual data points are protected^73^. However, it leads to huge loss of accuracy due to the heavy noise added at each client’s end^20^.

### Masking-based techniques

Masking techniques anonymize or pseudonymize data before sharing, reducing the risk of data reconstruction while maintaining its utility^74,75^.

- *Anonymization*: Involves removing or modifying personally identifiable information before data processing. In FL, anonymization ensures that client identities are not linked to their model updates, preventing targeted attacks^76^. However, it does not provide mathematical privacy guarantees, making it vulnerable to de-anonymization attacks if combined with external datasets^77–79^.
- *Pseudonymization*: Replaces personal identifiers with randomized or hashed values, such that it does not allow direct linkage to the individual participants^80^. Pseudonymization is weaker than encryption or DP in preventing adversarial reconstruction but helps mitigate inference attacks^81,82^.

### Tokenization

Translates sensitive data elements into non-sensitive equivalents (tokens) that have no exploitable value outside the training environment of a model. Within FL, this is often performed in conjunction with encryption to protect the metadata and stay compliant with other privacy regulations including GDPR and HIPAA^83,84^.

### Privacy-preserving techniques in FL: trade-off considerations

Each technique presented above offers a balance between privacy guarantees, computational efficiency, and communication overhead. The selection of a particular approach depends on the needs of an FL system:

- *Strength of security*: Techniques based on encryption such as SMPC and HE bring strong privacy guarantees but have computational overhead^23,85^.
- *Scalability*: Techniques based on differential privacy-DP and LDP-increase scalability but add noise-affecting model accuracy.
- *Lightweight*: Anonymization techniques and Tokenization are lightweight; they, however, lack cryptographic privacy promises^27^.

Hybrid approaches are the most typical ones in practical FL deployments. For instance, DP and encryption finds a balance in terms of balancing privacy preservation versus model performance; Federated averaging and homomorphic encryption can ensure privacy-preserving secure aggregation without exposing individual updates.

With the integration of these privacy-preserving techniques, federated learning can effectively protect sensitive data while enabling collaborative model training across distributed clients, particularly in privacy-sensitive domains like healthcare, finance, and personalized AI systems.

## Methodology

### Dataset and preprocessing

**Fig. 4:**
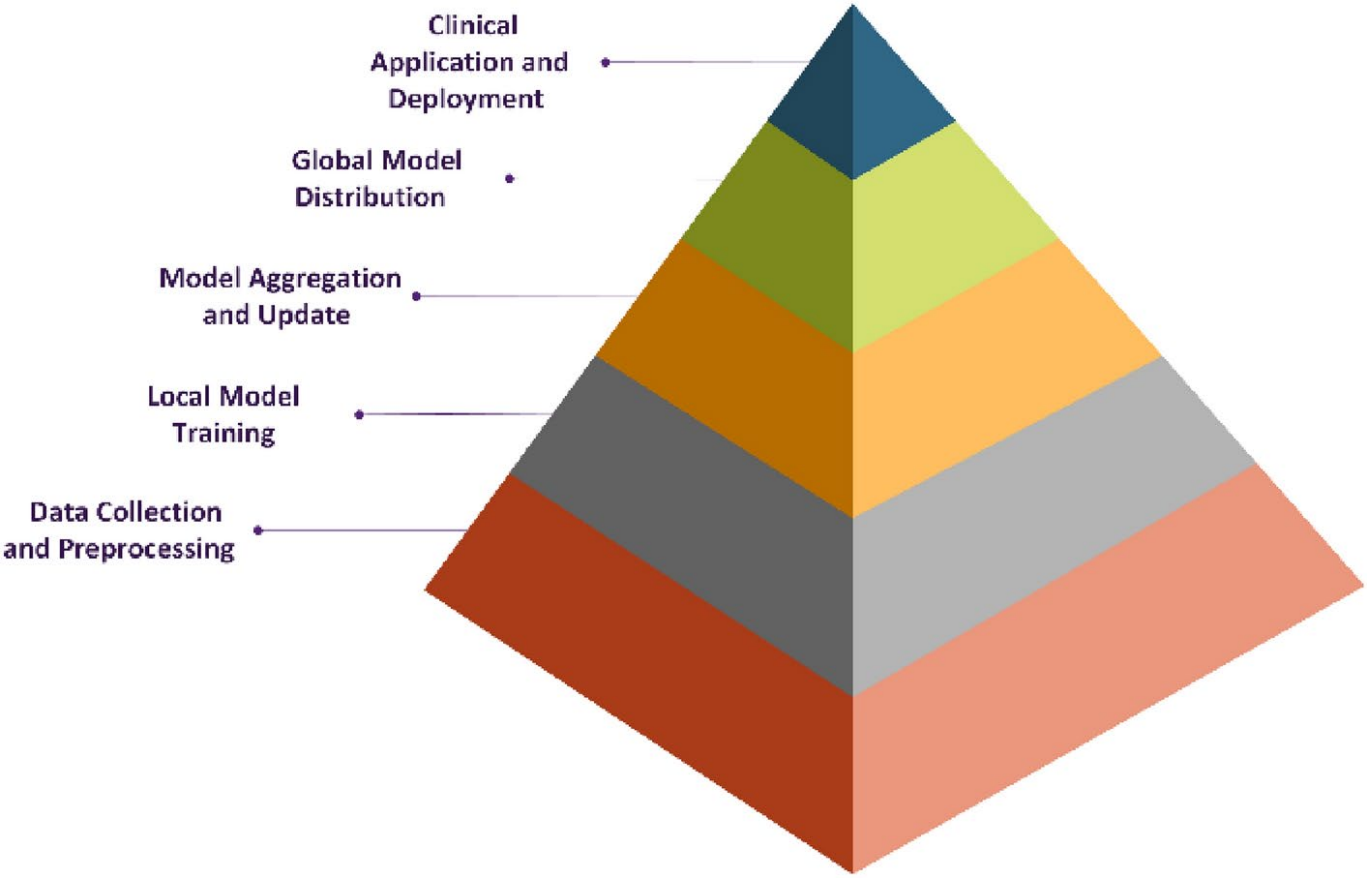
Federated learning in healthcare: A hierarchical workflow.

The successful integration of privacy protection measures in any healthcare area necessitates the implementation of federated learning. The hierarchical pyramid diagram shown in Fig. 4 visually represents the sequential stages involved in training and deploying a machine learning model, particularly in a federated learning (FL) setting. The base layer signifies *data collection and preprocessing*, where raw medical data is cleaned, normalized, and structured for model training. Moving upward, *local model training* occurs at individual client sites, ensuring privacy preservation by keeping patient data decentralized. The *model aggregation and update* phase consolidate locally trained models to form a globally optimized model, which is then *distributed back to clients* for further refinement. At the top, the *final model is deployed in clinical applications*, where it aids in breast cancer diagnosis. The structured progression highlights how FL ensures privacy, maintains performance across distributed datasets, and enables real-world medical integration.

### Dataset

**Fig. 5:**
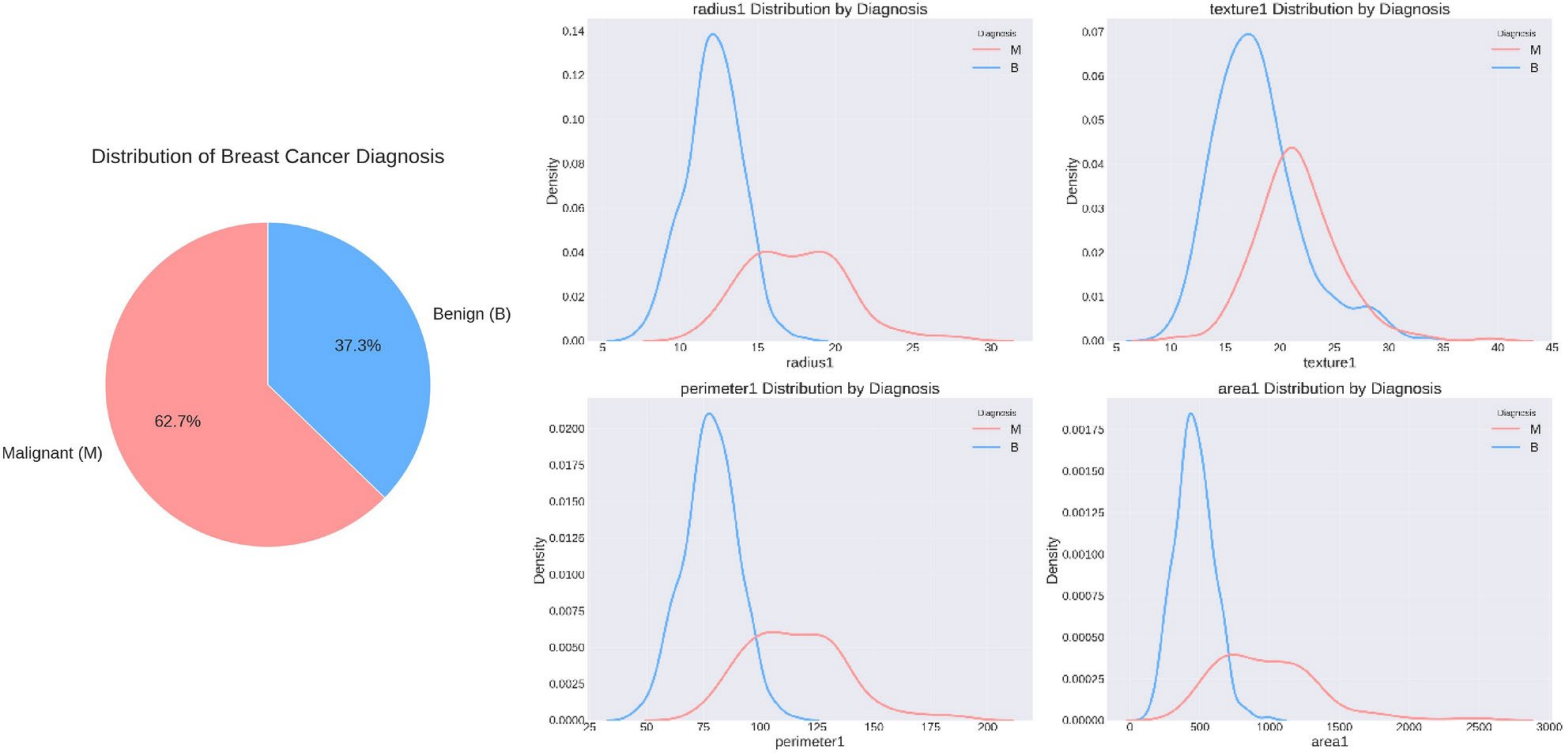
Overview of the breast cancer Wisconsin diagnostic dataset.

The Breast Cancer Wisconsin Diagnostic dataset, widely used in machine learning research, forms the foundation of this study. The dataset was obtained from the UCI Machine Learning Repository, a well-known source of benchmark datasets for medical diagnostics and classification tasks^24^. Table 2 and Fig. 5 present an overview of the Breast Cancer Wisconsin (Diagnostic) dataset. This set comprises around 569 instances of breast cancer tumors and has each observation defined by 32 features in this regard, which are reflections of different metrics obtained from breast cancer biopsies. Features include matrices that include the radius, roughness, smoothness, compactness, concavity, symmetry, and fractal dimension of cell nuclei. The diagnosis is indicated with a ‘B’ for benign and an ‘M’ for malignant. For analytical purposes, these categorical labels are translated into the binary format with 1 for malignant tumors and 0 for benign tumors. Consequently, the data set organized into 32 columns with one target column has resulted in a more comprehensive and improved data set for the evaluation of our model. The methodology of the study entails a few essential components. This was done to determine the level of accuracy and protection of privacy. Firstly, the dataset was used to build a machine learning model without integrating the federated learning method. Then, as a step towards enhancing the level of privacy, an implementation of a TensorFlow environment using the same dataset was done to begin federated learning with differential privacy.

**Table 2:** Breast cancer Wisconsin dataset overview.

Attribute	Description
Dataset name	Breast Cancer Wisconsin (Diagnostic)
Source	UCI Machine Learning Repository
Number of samples	569
Number of features	30 (e.g., radius, texture, perimeter, area, etc.)
Target value	Diagnosis (Malignant: 1, Benign: 0)

This research uses the publicly accessible Breast Cancer Wisconsin Diagnostic dataset, which does not include personally identifiable information. For real-time deployment of FL in clinical environments, however, ethical considerations must be addressed. Federated AI models learned from patient data need to comply with institutional review board (IRB) approvals and follow regulatory guidelines like HIPAA (Health Insurance Portability and Accountability Act) and GDPR (General Data Protection Regulation). Future deployments must have built-in explicit patient consent practices and ensure data governance policies keep up with the standards of ethical AI deployment within healthcare.

### Data preprocessing

**Fig. 6:**
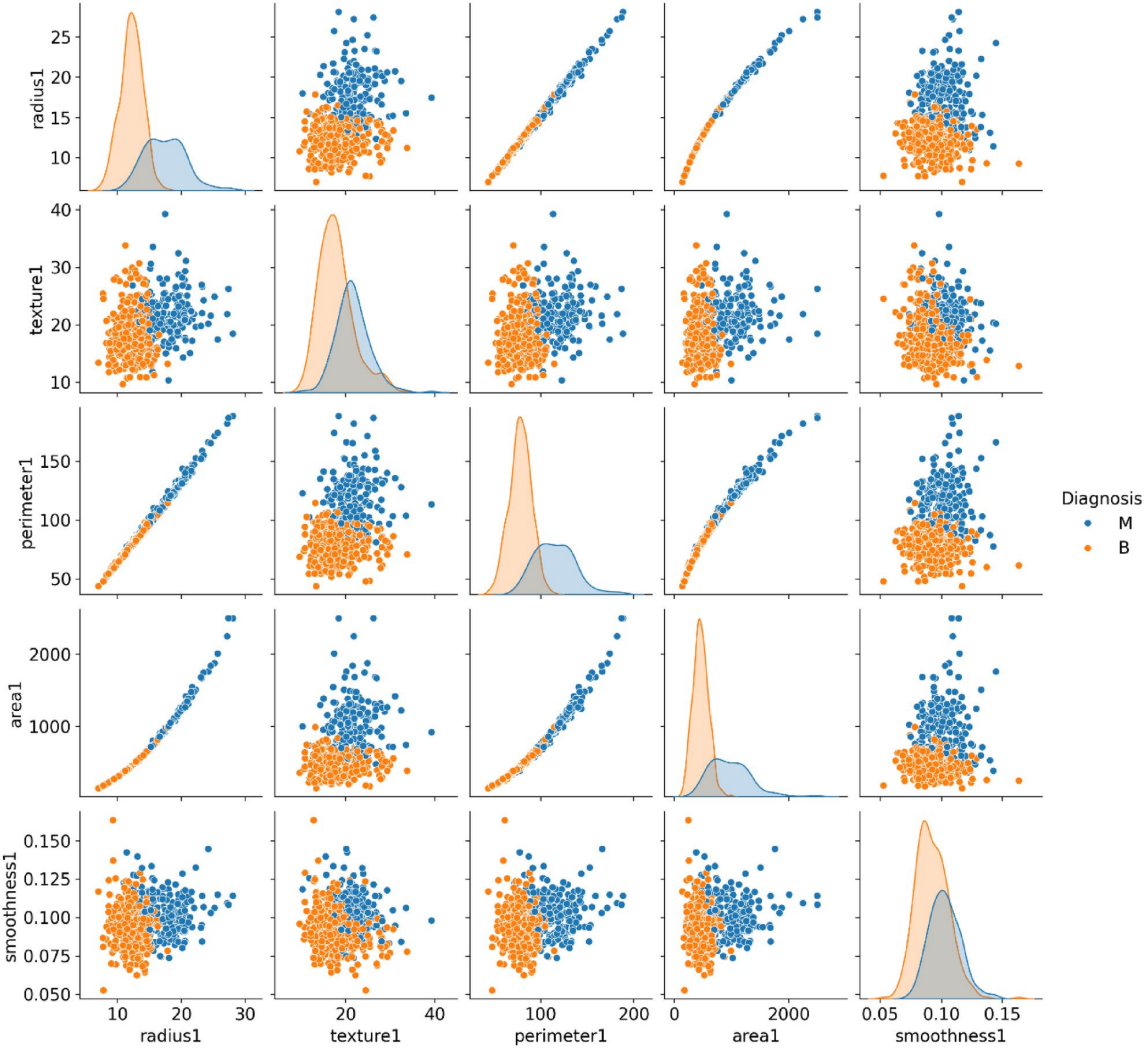
Pair plots illustrating feature distributions and relationships in the Breast Cancer Wisconsin Diagnostic dataset, differentiated by diagnosis (Malignant and Benign).

Preprocessing is a critical step in preparing the dataset for machine learning, ensuring data integrity, consistency, and proper feature representation. This study followed a systematic preprocessing pipeline that included handling missing values, feature scaling, encoding, feature selection, and dataset distribution strategies. Each step was carefully designed to optimize the dataset for federated learning while preserving privacy and improving model performance. Table 3 outlines the steps involved in data preprocessing, which include data cleaning, label encoding, normalization, and train-test splitting. These steps ensure that the dataset is properly formatted, standardized, and ready for model training and evaluation. The distribution of key features and pairplotting in the Breast Cancer Wisconsin Diagnostic dataset, differentiated by diagnosis (Malignant and Benign), is illustrated in Fig. 6.

**Table 3:** Steps for data preprocessing.

Step	Description
Data cleaning	Dropped unnecessary columns (Unnamed: 32, id) and handled missing values
Label encoding	Converted the Diagnosis column to binary values (Malignant: 1, Benign: 0)
Normalization	Applied Z-score normalization to scale features to a standard range
Train-test split	Split the dataset into 80% training and 20% testing subsets

#### Handling missing values

A thorough inspection of the dataset was conducted to identify any missing values, and it was confirmed that the dataset contained no missing entries. However, in real-world scenarios such as medical datasets, missing values are very common due to unfinished patient records, manual data entry errors, or missing diagnostic results. These missing values would be detected and handled by methods such as mean or mode imputation, removing all incomplete records. This step is very important since such discrepancies in one single client’s dataset could affect the global model’s performance. Model stability is enhanced and potential biases in learning are prevented if a clean and complete dataset is ensured before starting the FL training. In medical datasets, missing values would mean loss of valuable information, which could have contributed significantly to diagnosis^86^. To prevent these issues, standard imputation techniques such as K-nearest neighbor imputation or regression-based imputation are applied in scenarios where data loss is minimal, and estimation would be possible^87^.

#### Feature scaling and normalization

To normalize numerical attributes, feature scaling is essential so that all features serve equally to the learning process^88^. There are two different scaling techniques applied based on the type of model used:

- *Z-Score Normalization for Federated Learning (Neural Networks):*

To transform feature distributions to zero mean and unit variance Z-score normalization is used, calculated by the formula:

$$Z = \frac{ X - \mu }{\sigma }$$

Z: Represents the standardized value or Z-score, indicating how many standard deviations a data point *X* is from the mean *µ*. X: The original data point or observation from the dataset. *µ*: The mean (average) of the dataset, represented by the Greek letter mu (*µ*). *σ*: The standard deviation of the dataset, indicating how spread out the data points are from the mean, represented by the Greek letter sigma (*σ*).

This transformation prevents large numerical values from overpowering the learning process by ensuring that all features have similar scales^89^. This also balances model convergence, improves model training efficiency in federated learning models based on neural networks and minimizes the risk of exploding or vanishing gradients. This method is very advantageous in gradient-based optimization, where having uniformly scaled inputs improves weight adjustments and enhances backpropagation.

- *Min–Max Scaling for Tree-Based Models (Random Forest):*

This method is used to convert feature values into a fixed range of [0,1], which is more comprehensible for tree-based models. Min-Max scaling does rely on gradient descent optimization, but it relies on normalized feature ranges, which enhances decision boundary formation and makes sure that all the feature values lie within the same range, which allows the tree-based models to split decision nodes efficiently while minimizing the computational overhead. The formula given below represents the Min-Max Scaling technique, which is commonly used to normalize features within a specific range, typically [0, 1]. This scaling technique helps maintain the relationships between data points while ensuring that all features contribute equally to the model, which can be beneficial in certain scenarios, including ensemble learning with tree-based models.

$$X_{scaled} = \frac{{X - X_{\min } }}{{X_{\max } - X_{\min } }}$$

X_scaled_: Represents the scaled (normalized) value of the original data point after applying Min–Max Scaling. X: The original data point or feature value that needs to be scaled. X_min_: The minimum value of the feature in the dataset, used as the lower bound for scaling. X_max_: The maximum value of the feature in the dataset, used as the upper bound for scaling.

The scaling strategy used was determined by the differences in the way the models are designed to process the data concerned and which would thus bring out the highest performance in both FL and non-FL cases. Scaling is also an important part of the process since it guarantees that the model learning is not biased by the distribution pattern of the features, which is especially critical in medical applications where different features might have significantly different ranges by their innate qualities^89^.

#### Encoding the target variable

Since machine learning models require numerical inputs, the categorical diagnosis labels were transformed into binary values to facilitate computation^90^. The diagnosis labels ‘B’ (Benign) and ‘M’ (Malignant) were converted into numerical representations:

- Benign (B) → 0
- Malignant (M) → 1

This encoding is necessary for smooth recognition in machine learning programs and for the uniform way that the programmed exercise is presented. In binary encoding, the classification model can distribute weight effectively among different classes thus establish successful model optimization without overfitting to the training data, which is important for example, in logistic regression or neural network-based structures where loss functions are highly nonlinear. In federated learning, the maintenance of the same encoding in all the clients is essential to prevent the data inconsistency among the participants.

### Feature selection

**Fig. 7:**
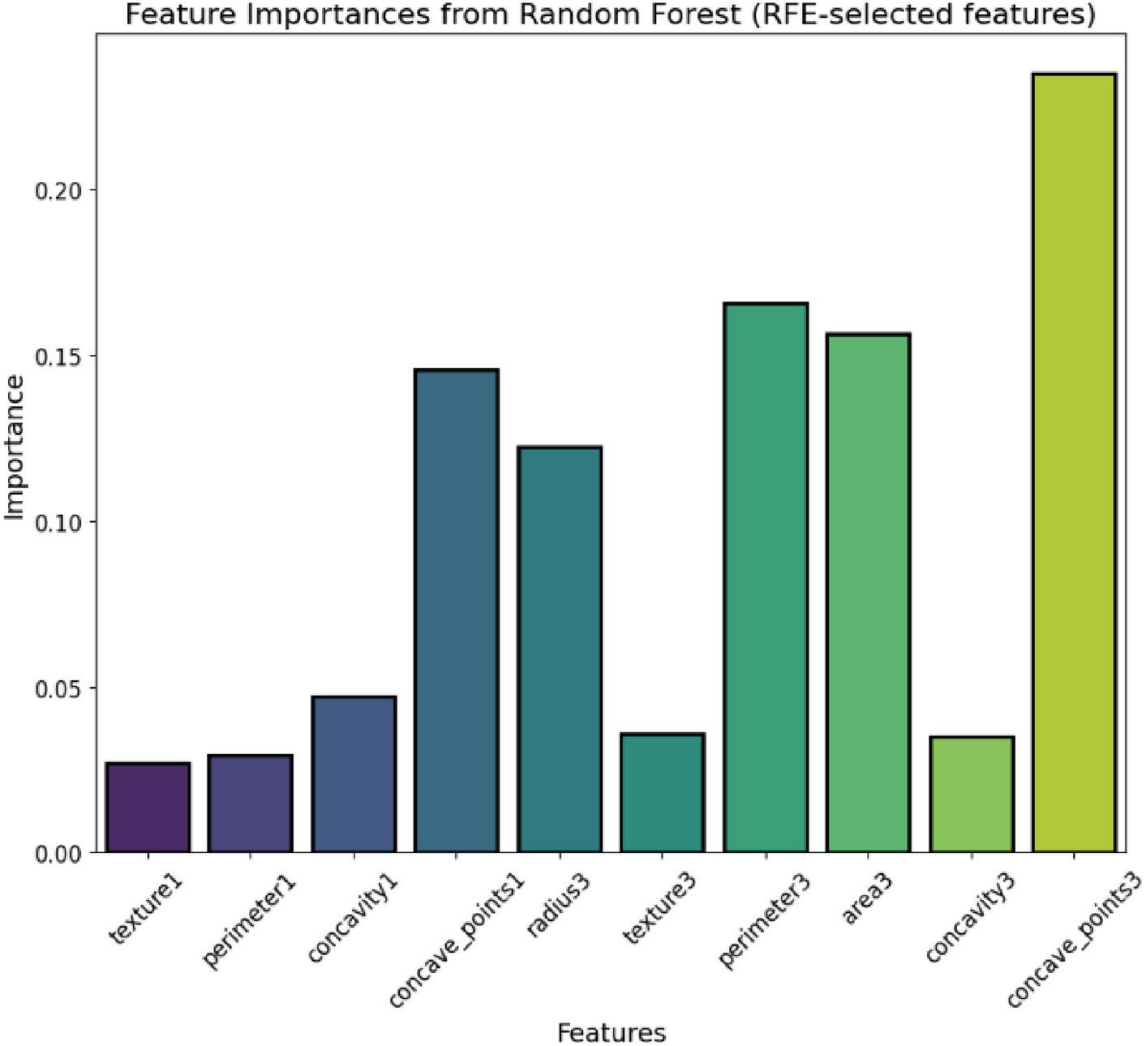
Feature importances from RFE.

**Fig. 8:**
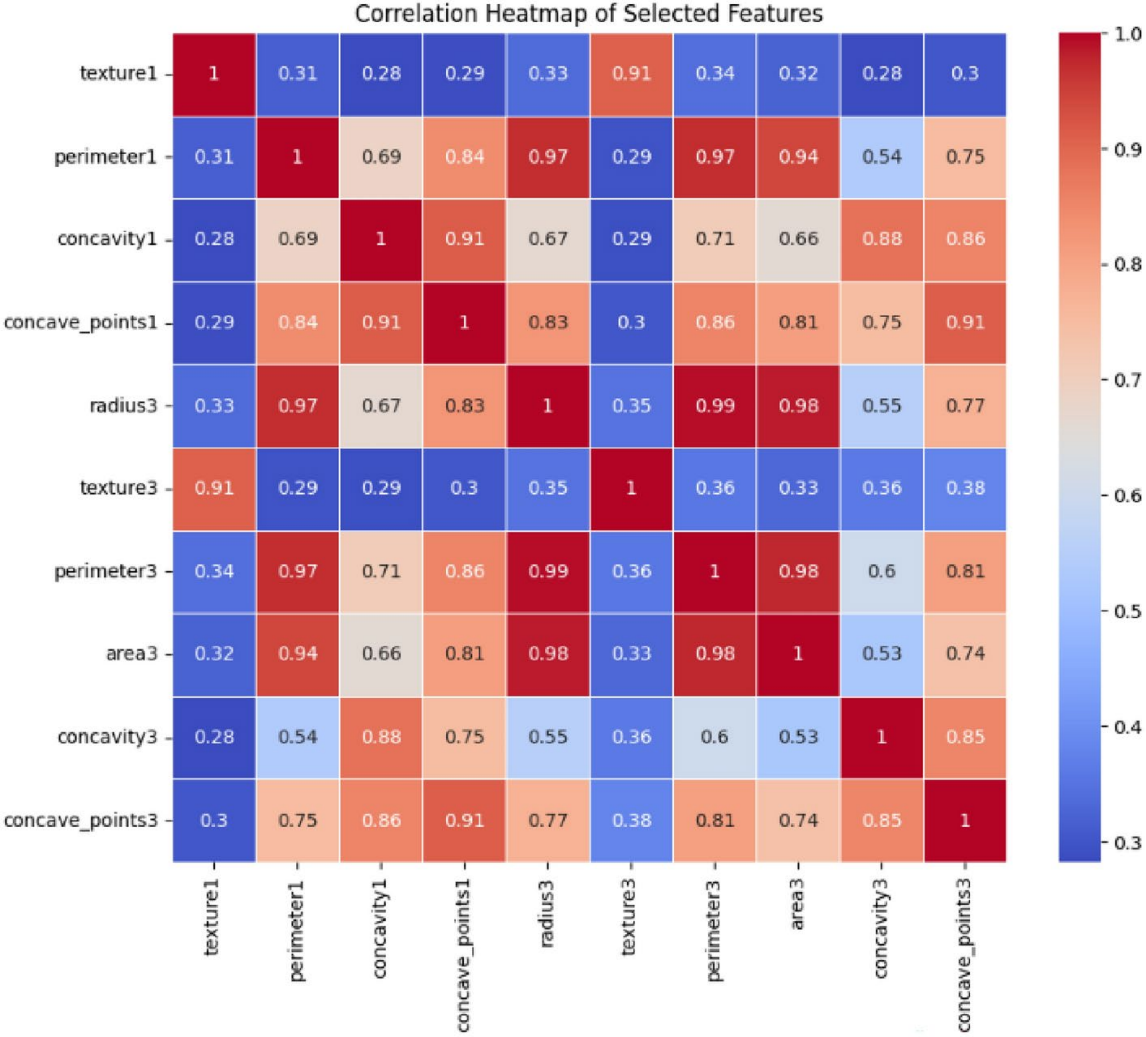
Correlation heatmap of selected features.

Feature selection is one of the critical operations in machine learning, which aims at discovering the most relevant and indispensable features for the model training to enhance performance and reduce the computational complexity of the model. Feature selection was performed to improve computational efficiency, reduce overfitting, and enhance model interpretability, ensuring that only the most relevant attributes contribute to classification accuracy^91,92^. RFE was used in the research to consider only those features that have been identified to be important. RFE is a feature selection technique in which the model iteratively filters out the least important feature while taking into consideration their effects on the training model through iterative training. Table 4 presents the feature selection process in detail. In the given scenario, RFE was combined with a Random Forest Classifier. Among the ensemble-based machine learning methods, Random Forest Classifier is the one best known for its accuracy and resilience while operating on high-dimensional data. In the process, RFE had included all features at the beginning and gradually removed non-important ones till only the most important were left. The major step of the process was fitting the random forest model on the training data. After this, features were ranked with the importance scores, and plots of correlation pairing were done; the top selected features that most significantly contributed to the improvement of the prediction in the model were chosen. The machine learning model of the dataset was trained using that final set of selected features. These are the two most major objectives of this selection method: first, which is to reduce overfitting; second, to focus majorly on the most important and informative features; and third, make the model interpretable by excluding elements that are irrelevant or redundant. The feature importances identified through Recursive Feature Elimination (RFE) and the correlation heatmap of selected features are presented in Figs. 7 and 8, respectively, providing insights into the dataset’s key attributes and inter-feature relationships.

**Table 4:** Feature selection.

Step	Description
Method used	Recursive Feature Elimination (RFE)
Selected features	Top 10 features selected based on importance
Estimator	Random Forest Classifier
Rationale	Reduce dimensionality and improve model performance

#### Federated learning dataset distribution

**Fig. 9:**
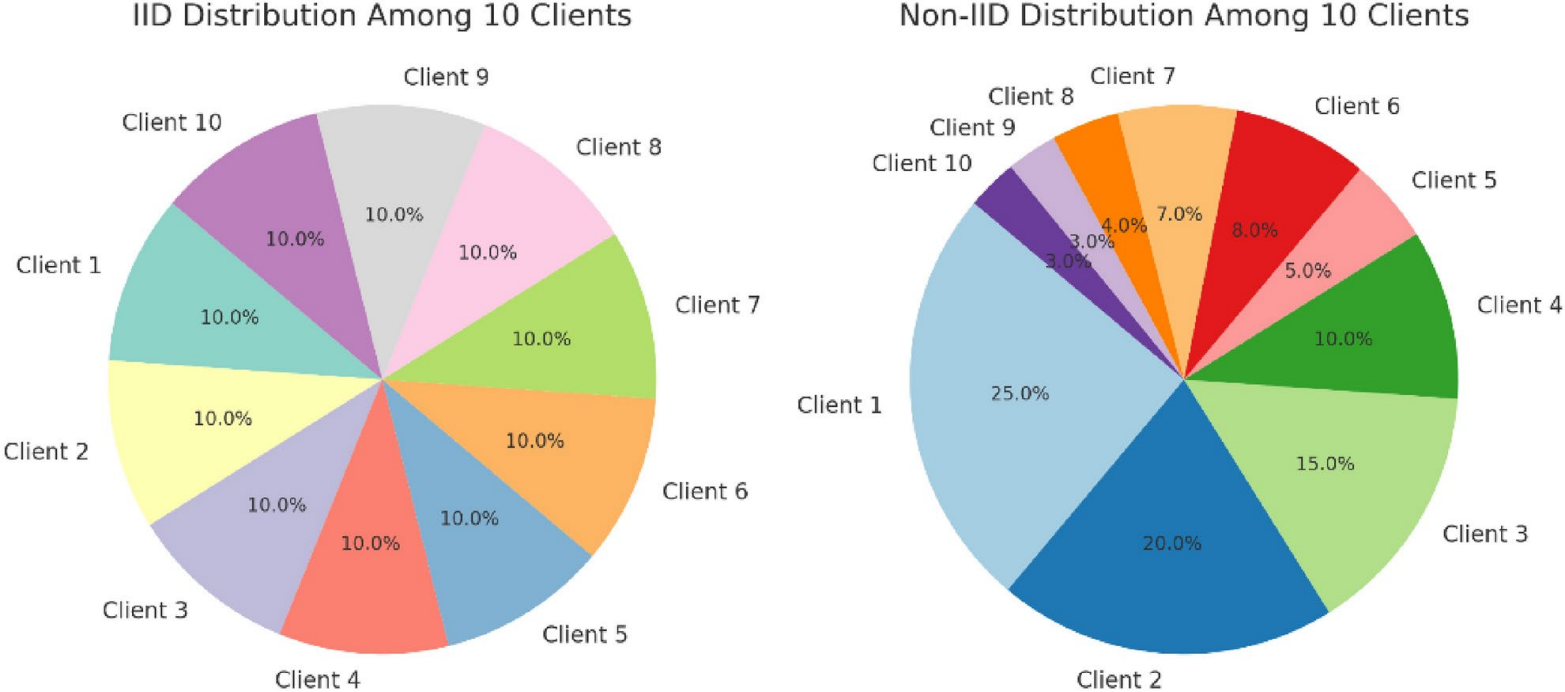
Distribution of data among federated clients.

To mimic a real-world federated learning setting, the dataset was shared with different clients according to the two approaches—IID (Independent and Identically Distributed) Configuration and Non-IID (Independent and Identically Distributed) Configuration. To accomplish IID (Independent and Identically Distributed) Configuration, the dataset was shuffled and split into parts at random equally for all clients. Each user got an equal amount of benign and malignant cases, meaning that the assignments were the same. This type of scenario was carried out in the relative performance measurement of the FL model in the case of the most optimal configuration^41^. For Non-IID (Non-Independent and Identically Distributed) Configuration, the dataset was unevenly allocated, modelling real hospital setting where disease prevalence differs from one hospital to another^93,94^. Some clients were given datasets containing mostly benign cases, while others received mostly malignant cases. This situation was done to see if the FL model is strong enough in classifying data that is not uniformly distributed across different clients without a central aggregator. A Fig. 9 the distribution of data among federated clients illustrates the difference that exists in the two approaches—IID and Non-IID. This step was crucial in determining how well the FL model can distinguish medical datasets that are diverse in nature.

### Random forest model architecture and training without federated learning

**Fig. 10:**
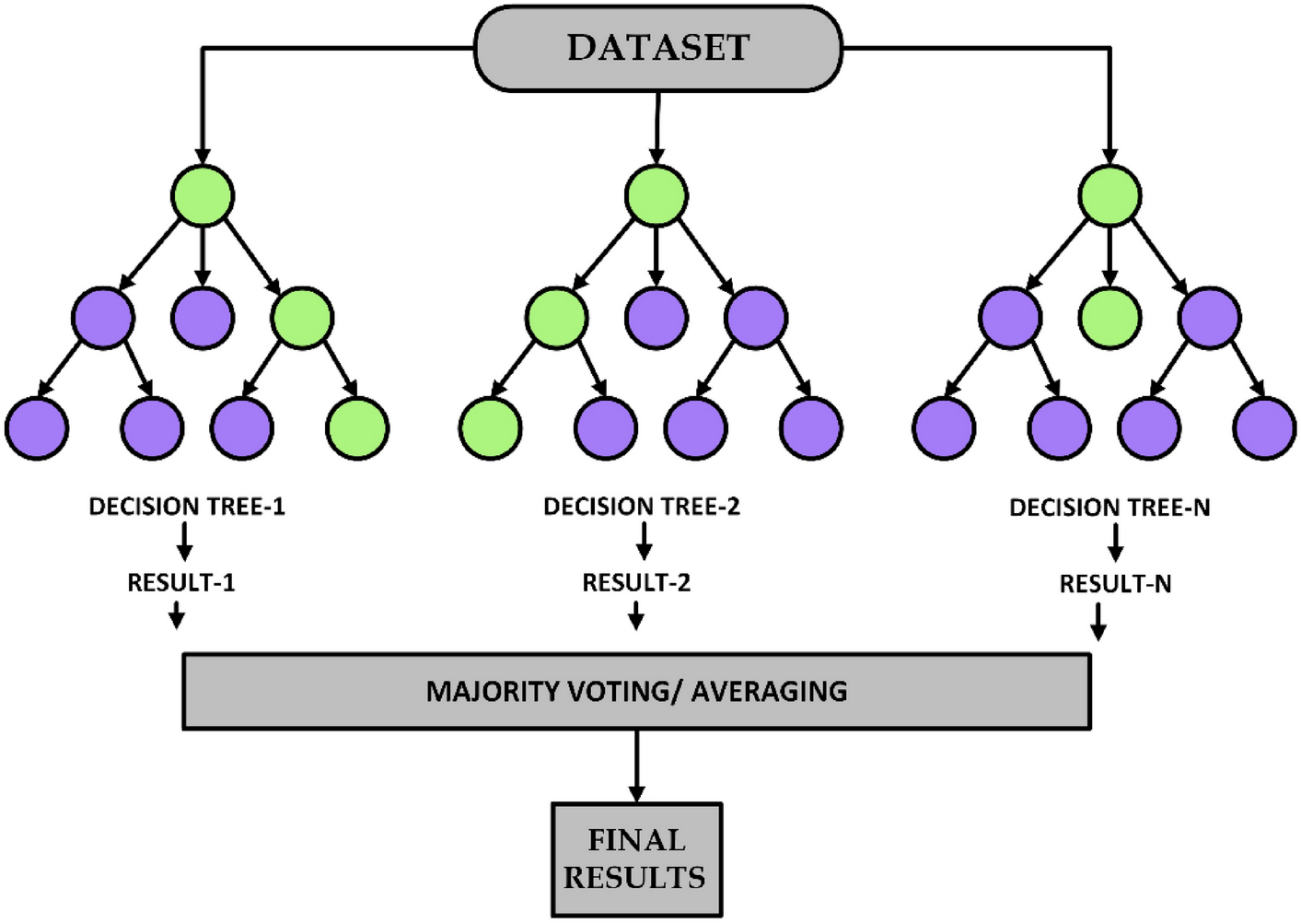
Random Forest architecture.

The Random Forest classifier is an ensemble learning approach that optimizes the classification accuracy of the decision tree by creating various decision trees and combining their predictions. In contrast to the overfitting-prone singular decision trees, Random Forest unites numerous models thus a more widely adopted and strong classification output is attained. This feature is especially significant for medical diagnosis where wrong classification may bear great implications^95^. The architecture of the ensemble learning method using multiple decision trees with majority voting or averaging for final predictions is illustrated in Fig. 10.

#### Random Forest architecture and functionality

The Random Forest technique comprises training numerous decision trees on distinct data samples which are collected from the dataset through the bootstrap sampling technique. Each tree functions as a separate entity. Hence, it is decided that the most attended tree by all is to be the one, which is used in the classification process^96^. The main benefit of the Random Forest algorithm is the ability to process data with a high dimension, reduce the variance of the overall result, and stabilize the model. The figure below represents a detailed interface of the Random Forest^97^.

- *Decision Tree Construction*: The Random Forest is made up of numerous different data subsets, each of which is a decision tree built from the training data. Ensemble methods are typically employed when creating a tree that analyze the multiple decision paths resulting from a split of the dataset. Nodes are the feature splits, and leaves are the outputs of the final classification^98^.
- *Feature Subset Selection*: At every tree split, only a small contributor group of the features is used for determining the best split, which in turn helps to prevent the trees from becoming too similar. The trees being not like each other like in this case is crucial for generalization improvements^99^.
- *Voting Mechanism*: The process of making predictions involves each of the decision trees examining the input sample without the participation of other trees. The majority voting of all trees that someone has a sample to be classified leads to the final decision. This makes the system robust against overfitting and ensures that generalization is not a problem^100^.
- *Handling Class Imbalance*: The Random Forest algorithm collects class samples from the training data in such a way that an approximate even division of all classes is achieved^101^. In other words, the Random Forest algorithm gives more importance to the instances of the minority class, and thus, it can be used to prevent bias towards the majority class.

A feature of Random Forest that is very useful in a medical diagnostics context is its capacity to present the user with the relative importance of all features, making it clear what features influenced the decisions. The interpretation of this part is extremely important in clinical settings, as it allows domain experts to check the decisions taken by the machine against standard diagnostic markers.

#### Advantages of Random Forest for breast cancer classification

The popular Random Forest algorithm for machine learning has several key advantages. The main difference is that, unlike the overfitting caused by individual trees that commit to training data, Random Forest uses the average of many different tree predictions to help it avoid overfitting, thus improving generalization and decreasing model variance. The method also guarantees high predictive accuracy, often exceeding that of individual classifiers. Moreover, because of the high degree of scalability, the model efficiently handles high-dimensional and large datasets without putting much strain on the computer resources. Another one of its most remarkable characteristics is that it is very resistant to noise in the data; instances that are outliers and noisy examples will carry very little weight in the resulting predictions, as every tree processes only a portion of the data. In addition, Random Forest can produce useful knowledge through feature importance analysis systems; in this way, it can identify the top features based on their predictive power. Interpretability of the model remains crucial, especially when used in areas that require a responsible occupation such as in medical applications.

#### Random Forest training pipeline

The procedure to train the random forest classifier is to start with several key procedures. The process started with preprocessing the dataset, all the features including feature selection, normalization, and encoding thus the data is to be ready for modelling. It was during the bootstrap sampling of the training stage that chunks of training data were created with random selections which were inspired by the healthy distribution idea. When the forest was made, each tree was trained independently doing the same process again but, this time a different subset possibility was considered to ensure diversity between the trees. After the model was trained, the model was tested on the test set by the method of majority voting that was initiated across the trees. To ensure stability and robustness in classification, majority voting was applied to the trees’ predictions generated on the entire test dataset. The finished classifier was then verified with a confusion matrix, a classification report, and a feature importance analysis, along with its classification accuracy and interpretability assessments.

#### Hyperparameter selection and model configuration

The hyperparameter tuning process was carried out systematically by adjusting key parameters to enhance model performance. Empirical validation played a crucial role in optimizing these hyperparameters to achieve higher predictive accuracy^102^. In the Random Forest model, the number of estimators was set to 100, meaning 100 individual decision trees were formed. A higher number of trees reduces variance, stabilizing predictions while preventing excessive sensitivity to training data. The maximum depth of each tree was limited to 10, preventing excessive growth that could lead to overfitting while ensuring that the model generalizes well to unseen data^103^. To further control model complexity, the minimum split was set to 4, ensuring that a node must contain at least four samples before splitting, thereby preventing unnecessary divisions. Similarly, minimum samples per leaf were defined as 2, meaning that at least two samples were required in a leaf node for statistically significant decisions, reducing the risk of overfitting. The maximum features considered for each split was set to the square root of the total number of features, ensuring diversity in tree structures and reducing correlation between them.

Table 5 presents the selected hyperparameters and their corresponding values used in the Random Forest model. Each parameter plays a crucial role in optimizing model performance, preventing overfitting, and ensuring generalization.

**Table 5:** Hyperparameter selection and model configuration.

Hyperparameter	Value
Number of Estimators (n_estimators)	100
Maximum Depth (max_depth)	10
Minimum Samples Split (min_samples_split)	4
Minimum Samples per Leaf (min_samples_leaf)	2
Maximum Features Considered (max_features)	sqrt
Bootstrap Sampling	Yes

In addition to these hyperparameters, bootstrap sampling was used, where each decision tree in the Random Forest model was trained on a randomly selected subset of data points with replacement. This technique ensured that different trees learned distinct patterns from the dataset, leading to a more generalized model while minimizing overfitting. To further refine hyperparameters, Grid Search Cross-Validation was employed, identifying the most effective candidates through extensive validation. The final model was validated using training data to guarantee robust performance when applied to unseen datasets.

#### Model training

**Fig. 11:**
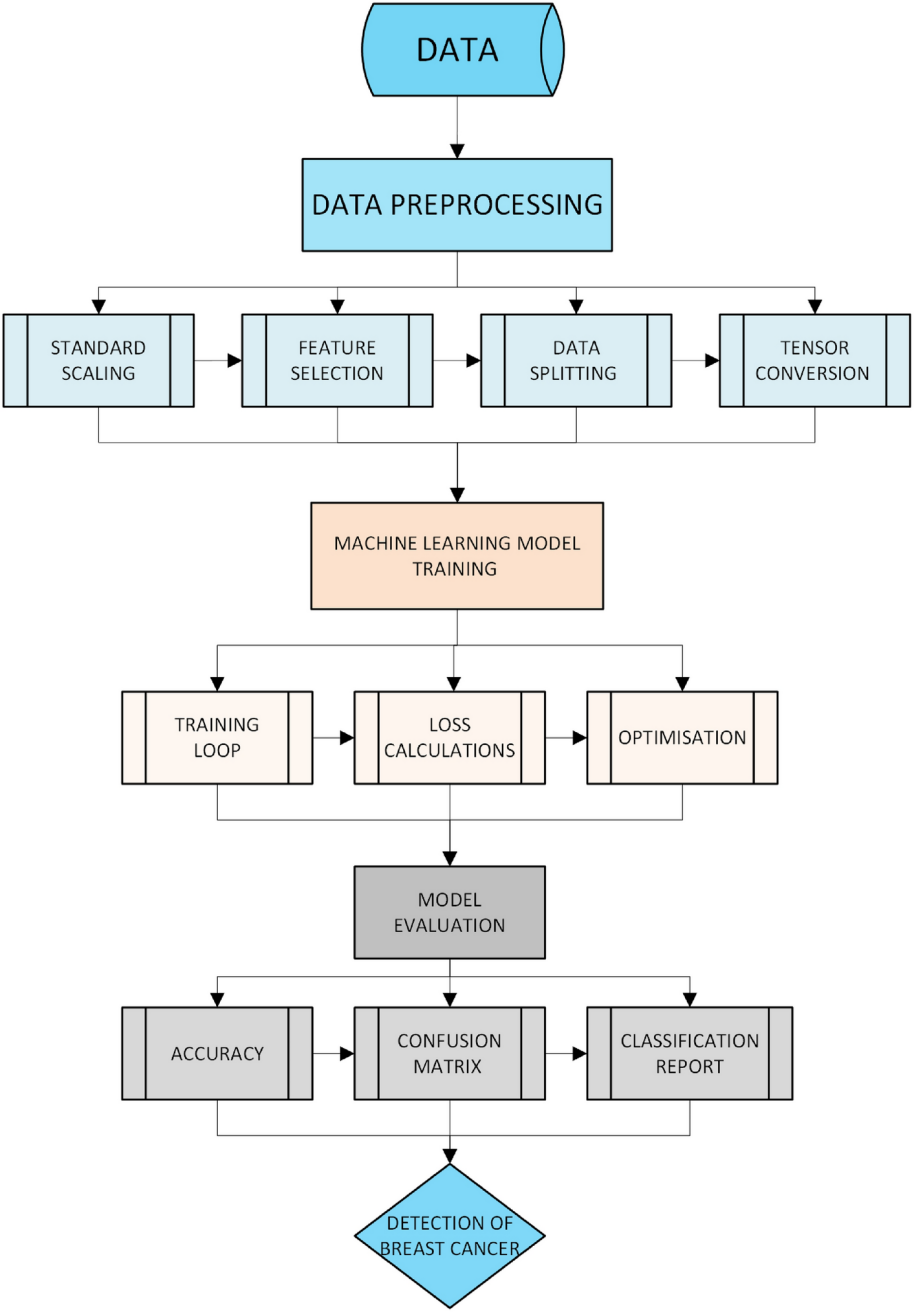
Machine learning without FL implementation methodology flowchart.

The workflow of the machine learning pipeline for breast cancer detection without FL, including data preprocessing, model training, evaluation, and final prediction, is illustrated in Fig. 11. This starts with data preprocessing, which includes the practices of standard scaling, feature selection, data splitting, and tensor conversion, necessary to set the dataset ready for the training part. The training section will be next, which typically consists of such steps as a training loop, loss value evaluations, and optimization for better model parameter fitting. At the end of training, it will be followed by the evaluation, in which performance indicators like accuracy, confusion matrix, and classification reports are outputted. Finally, a human gets to the breast cancer detection part, and the possibility of the use of the trained model in clinical decision-making is demonstrated.

The dataset was split into an 80–20 train-test partition, where 80 percent of the data was allocated for training and 20 percent was reserved for evaluation. This split ensured that the model was trained on a sufficiently large dataset while leaving an independent test set to assess performance on unseen samples. The training process involved the construction of 100 decision trees, each independently trained on a bootstrapped subset of the data. Once trained, these trees collectively determined the class label of each test instance through majority voting, thereby reducing the likelihood of incorrect classifications caused.

### Federated learning model (FL) training

**Fig. 12:**
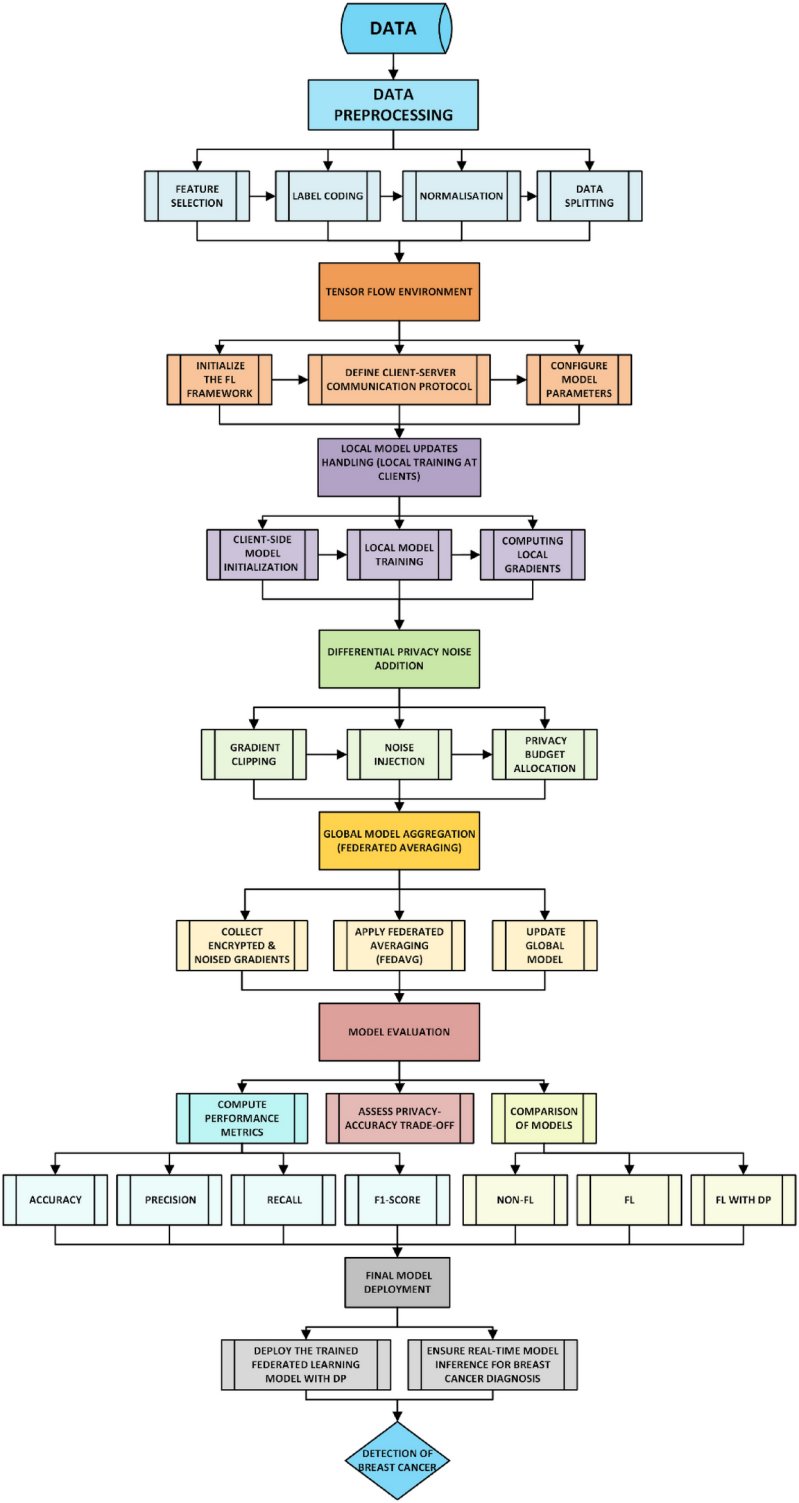
Machine learning with FL implementation methodology flowchart.

Federated Learning (FL) represents a decentralized machine learning methodology in which various clients can work out a wide application of the model together without being obliged to open their internal datasets. This type of approach is particularly important in the field of healthcare, especially in breast cancer diagnosis, where patients’ data privacy and regulatory constraints limit the centralization of sensitive patient data. The present study adopted the implementation of FL by means of TensorFlow Federated (TFF) which enabled secure distributed training across ten clients, each of whom was a medical institution with its own patient data. The implementation methodology for machine learning with federated learning is illustrated in Fig. 12. The implementation of FL via TensorFlow Federated (TFF) was performed in such a way as to provide that each client independently trained its model and informed the central server only of its model updates (gradients and weights) with the help of the client. The major steps of FL implementation were:

- *Client Initialization*. The dataset assigned to each client was proportional to either IID (Independent and Identically Distributed) or Non-IID (Non-Independent and Identically Distributed). In the IID setting, each client received a random and balanced subset of benign and malignant tumor cases, thus reaching a uniform representation. The non-IID setup was in full conflict with the IID situation with the real scenario in which different medical institutions had different distributions of cancer cases.
- *Local Model Architecture*. The client trained the fully connected feed-forward neural network (FNN) that is not more than a copy of the global model and then sent the same update to the global model. The FNN was characterized by the specific architecture including:

*Input Layer:* 30 neurons corresponding to the 30 numerical features in the dataset.

*Hidden Layer:* 16 neurons with ReLU activation, enabling the experiment on the data.

*Output Layer:* 1 neuron with sigmoid activation, executing binary classification (benign vs. malignant). Such architecture ensured a proper balance between processing capability and identification accuracy.

Table 6 presents the architecture of the fully connected feed-forward neural network (FNN) used for local training in the Federated Learning setup.

**Table 6:** Local model architecture in federated learning.

Layer	Description
Input layer	30 neurons corresponding to the 30 numerical features in the dataset
Hidden layer	16 neurons with ReLU activation
Output layer	1 neuron with sigmoid activation for binary classification (benign vs. malignant)
Optimizer	Stochastic Gradient Descent (SGD) with a learning rate of 0.02

- *Local Training Process*. Clients were trained by the clients on their own local models using Stochastic Gradient Descent (SGD) with a learning rate of 0.02. Each client executed numerous training epochs by modifying local model weights before sending them to the server. The training was unique and was performed on the dataset of each client. Also, no raw patient data was exchanged.
- *Model Updates Transmission*. The local clients exchanged only model gradients and model weights with the central server after training on their local dataset. Consequently, there was no sharing of actual patient data in this instance, thereby significantly augmenting privacy.
- *Global Model Aggregation*. Using Federated Averaging (FedAvg) The local model updates were then aggregated by the central server using the Federated Averaging (FedAvg) algorithm.
  The choice of using FedAvg over FedSGD was made due to its provision of multiple local updates prior to server communication, which in turn led to reduced bandwidth usage and improved convergence. Largely, even though the Federated Stochastic Gradient Descent (FedSGD) testing was carried out, it indicated the model performance’s high variance and hence the FedAvg was selected as the best approach. Table 7 compares the Federated Averaging (FedAvg) and Federated Stochastic Gradient Descent (FedSGD) algorithms, highlighting why FedAvg was chosen for the study.

**Table 7:** Comparison between FedAvg and FedSGD.

Feature	FedAvg	FedSGD
Local updates performed	Yes (multiple per round)	No (only one per round)
Bandwidth usage	Lower	Higher
Convergence stability	More stable	Higher variance in updates
Communication frequency	Less frequent	More frequent
Preferred for FL?	Yes	No

- *Model Synchronization and Iterative Training*. The server sends the clients an envelope with a copy of the new global model, which they open by the latest local training procedure in subsequent rounds. This process of iteration was repeated until the model did arrive at a certain change point.

Table 8 outlines the key steps involved in Federated Learning, detailing the process from client selection to global model synchronization.

**Table 8:** Key steps in federated learning workflow.

Step	Description
Client selection	Randomly selecting a subset of clients for each training round to optimize computation efficiency
Local model training	Clients train their models independently on local datasets
Gradient clipping	Restricting gradient magnitudes to prevent excessive influence from single data points
Differential privacy (DP)	Adding Gaussian noise to gradients to ensure privacy preservation
Model update transmission	Clients send noise-protected model updates to the central server
Global model aggregation	Server aggregates all updates using the Federated Averaging (FedAvg) algorithm
Model synchronization	Updated global model is redistributed to clients for further training

#### Differential privacy architecture for federated learning

**Fig. 13:**
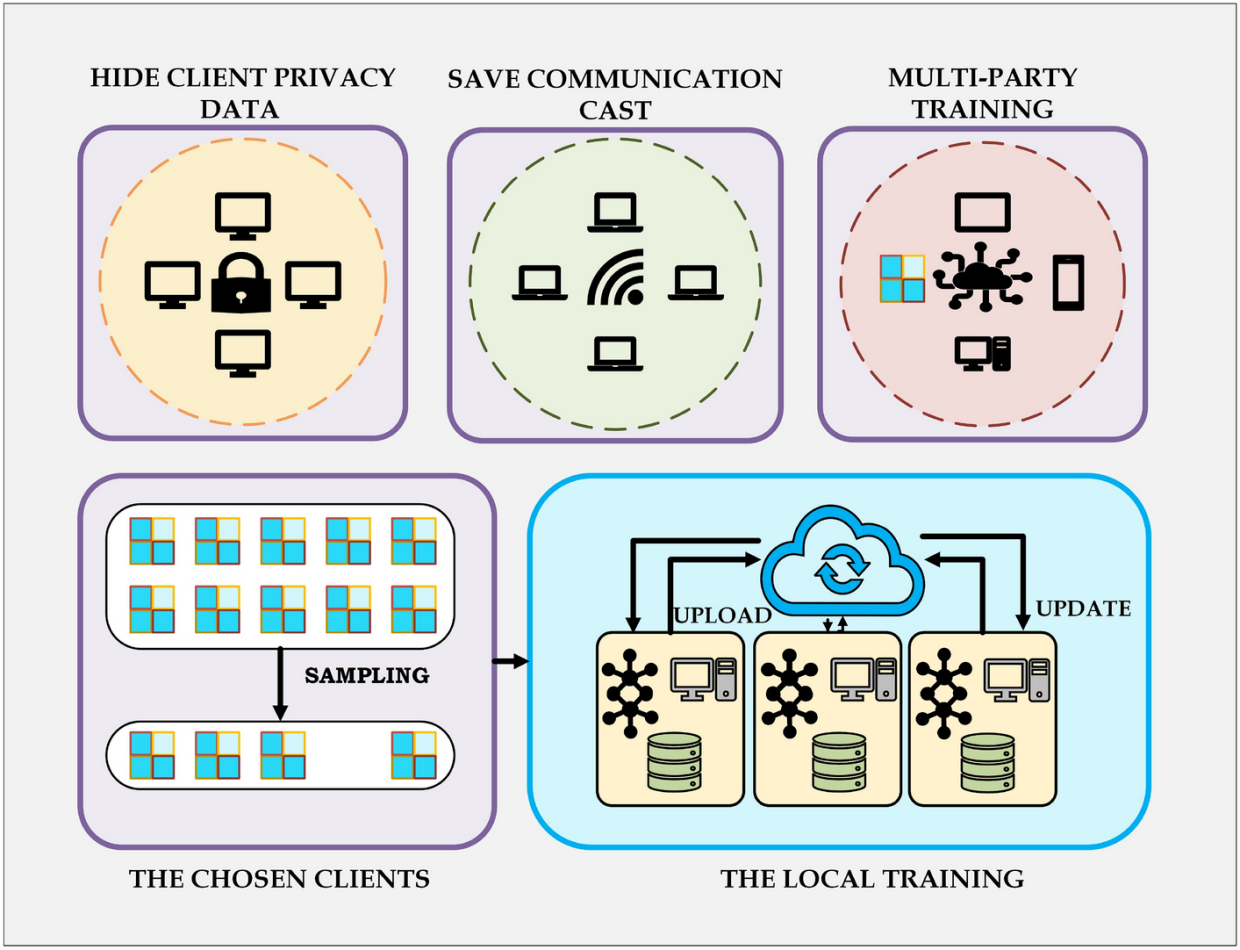
DP architecture.

The architecture of differential privacy in federated learning, highlighting client data privacy preservation, communication efficiency, multi-party training, client selection, and local model updates, is illustrated in Fig. 13. The implementation of Differential Privacy in the framework of Federated Learning is a means of suppressing sensitive information on clients by preventing model updates from being too heavily influenced by individual data points thus thwarting the efforts of malicious attackers to uncover private data^104,105^. In simpler terms, differential privacy ensures that individual patient data cannot be identified even when participating in training. The privacy budget (ε) controls the amount of noise introduced into data; smaller values guarantee better privacy but can affect model performance. One good analogy is to decrease background noise on a phone call—excessive noise (low ε) makes the call difficult to hear (model predictions), but insufficient noise (high ε) could jeopardize privacy. The δ parameter controls the possibility that privacy bounds may be marginally broken while offering a manageable trade-off between security and model utility. The system can be analyzed in three distinct areas: namely the client privacy protection, the communication efficiency, and the secure training among multiple parties that employ the globally aggregated data and users the places with which they are directly connected to themselves^106^.

#### Key components of differential privacy in FL

*Privacy Protection for Clients*. The main goal of implementing DP in FL is to ensure privacy. An example of this is presented in the section on hiding client privacy data in the figure, where all clients have their local datasets to work on and do not share raw data with anyone, including the central server. The only information exchanged consists of model updates (gradients and weights)^7^.

Clients build models in their local environments using their own data, preventing direct access to sensitive medical records. Before transmitting model updates, each client applies gradient truncation, limiting the influence of single data points by trimming the largest gradient values. This step safeguards against adversaries attempting to infer individual contributions to the model. To further enhance privacy, Gaussian noise is added to gradients before they are sent to the central server. This ensures differential privacy guarantees by obscuring the contribution of any individual data point. The approach outlined above significantly reduces the risk of membership inference attacks, where an adversary attempts to determine whether a particular record was included in the training dataset, thereby preventing unauthorized association with the training data^107^.

*Communication Efficiency in Federated Learning*. The communication cost section of the architecture highlights the importance of distributed-sharing concerns, which are addressed by differential privacy to enable federated training with low communication overhead. In conventional federated learning, each client updates its model in every round, leading to excessive network traffic and making the learning process inefficient. To mitigate this, differential privacy mechanisms are employed to optimize communication efficiency^108^.

One approach is reducing gradient sensitivity, where the variance of gradients is minimized compared to other methods, ensuring a more stable training process. Additionally, efficient secure aggregation is implemented to safeguard the most sensitive parameters, allowing gradients to be shared in an aggregated or non-aggregated manner but with high noise, reducing the risk of data leaks. Another crucial aspect is minimizing round communication, achieved by introducing randomness into the model parameters through differential privacy. This helps the model reach convergence with fewer communication rounds, significantly enhancing training efficiency.

These optimizations collectively strengthen the robustness of Federated Learning models while maintaining a high level of privacy^7^.

*Secure Multi-Party Training in FL*. The multi-party section in Federated Learning (FL) represents the decentralized nature of the framework, where different clients collaborate without directly exchanging raw data. Secure training is ensured through several mechanisms.

In decentralized learning, clients independently update their models and send only noise-protected centralized updates to the server, preventing direct data exposure. Additionally, adaptive privacy mechanisms allow dynamic adjustments of the privacy budgets (*ε, δ)* in accordance with the Differential Privacy (DP) method. The privacy budget determines the amount of noise added to updates, ensuring that a lower *ε* value provides higher privacy while maintaining model accuracy. Furthermore, FL adheres to regulatory compliance, ensuring that privacy regulations such as HIPAA and GDPR are met. This is particularly crucial for industries dealing with sensitive data, such as medical applications like breast cancer diagnosis^25^.

#### Workflow of differentially private federated learning

The bottom half of the architecture illustrates the FL training procedure combined with DP. This workflow covers not only one but several points in the process. These are:

- *Client Selection (Sampling)*. At each round of the training, some of the clients will be randomly chosen from the whole pool, which will decrease the computational burden and still have enough samples. This way, all clients do not need to take part in each round and get the same result, resulting in the training process being more effective.
- *Local Model Training at Clients*. The chosen clients conduct the training on their own data, which further ensures that computations are done in parallel across the network. Additionally, to improve privacy and security, gradient clipping is employed to restrict the sensitivity of each update to prevent extremely large gradients from strongly affecting the model. Furthermore, Gaussian noise is added to scramble individual contributions, which in turn additionally guarantees the privacy of client data while the training is ongoing.
- *Uploading Encrypted Updates*. Users send their secrecy-protected model updates to the server in the cloud. The gradients that are sent to the server remain anonymized. Consequently, there is no data that could lead anyone to identify the real identity of the patient, and in this way the data privacy and security are still maintained in the process of training.
- *Global Model Aggregation*. The main server overseas combining all the received updates into one which is done using the Federated Averaging (FedAvg) method, which is an approach that includes the contributions of all clients. These updates are still privacy-preserving, while remaining statistically useful owing to the Differential Privacy (DP) method that is applied to the sent data. The continuing modification of the global model through the rounds keeps the privacy of the data and the model accuracy in a balance.
- *Model Update and Redistribution*. Having updated the model, it is aggregated and sent back to the customers for continued, local training. The model formed in such a way goes through the process of iteration and becomes completely convergent, i.e. the correlation between privacy and the quality outcome of the model is achieved.

#### Privacy budget and its impact on model performance

Privacy budget (*ε*) is a crucial parameter in differential privacy, determining the balance between privacy protection and data utility. Besides affecting privacy loss, the privacy budget (*ε*) dictates the number of queries that can be tolerated. A lower *ε* value signifies greater privacy, though it may introduce higher vulnerability to model performance. In terms of configuration, an *ε* value of 1.0 offers moderate privacy while maintaining a balance between accuracy and security. Additionally, setting *δ* = 10^−5^ ensures a low probability of privacy compromise, strengthening the overall security of the model^109^.

In healthcare, striking the right balance is critical—a small privacy budget (strong privacy) may slightly lower diagnostic accuracy but ensures patient data security, while a relaxed budget prioritizes model performance at the cost of increased privacy risk. The impact of the privacy budget on model performance is evident in its trade-offs. Without differential privacy (DP), the model achieves high accuracy but lacks strong privacy safeguards. When DP is applied, a slight accuracy drop occurs due to the introduction of noise; however, the model remains privacy-preserving and resilient against data reconstruction attacks. To illustrate this effect, a privacy-accuracy trade-off graph was plotted, demonstrating that well-optimized privacy budgets can uphold diagnostic reliability while ensuring robust privacy protection.

#### Differential privacy implementation in federated learning

Federated Learning (FL) ensures that raw data remains physically on user devices, reducing direct privacy risks. However, when the model updates, personal privacy may still be at risk. Hackers could potentially attempt to reconstruct local training data by analyzing gradient updates. To mitigate this threat, Differential Privacy (DP) is integrated into FL by adding obfuscating client components, ensuring that individual contributions remain untraceable.

DP is applied by introducing Gaussian noise to gradient updates before they are sent to the central server. This mechanism ensures that individual observations remain hidden within the global model, preventing direct exposure. The implementation of DP in FL consists of the following key operations:

I. *Gradient Clipping*
  Before adding noise, gradient clipping is performed to limit the magnitude of updates. This step prevents outliers from disproportionately influencing the model, thereby enhancing stability and privacy.
II. *Noise Injection via the Gaussian Mechanism*
  After clipping, each client adds noise derived from a Gaussian distribution to its gradients. This process ensures that even if an attacker gains access to gradient updates, it remains difficult to distinguish individual client contributions, thus enhancing data privacy.
III. *Privacy Budget Allocation and Sensitivity Control*
  The privacy budget, represented by *ε* (epsilon) and *δ* (delta), determines the level of privacy protection. *ε* controls the trade-off between privacy and model utility, while *δ* ensures the probability of privacy breaches remains extremely low.
IV. *Federated Learning with DP Integration*
  Initially, users train their local models on-device. At the end of each training round, gradients are clipped to prevent excessive updates, and Gaussian noise is added before transmission to the central server, enhancing privacy. The global model is then updated using the Federated Averaging (FedAvg) algorithm, incorporating differentially private updates to balance model performance and privacy.

The FedAvg algorithm is given by:

$$w_{t + 1} = \mathop \sum \limits_{k = 1}^{K} \frac{{n_{k} }}{N}w_{k}^{t}$$

where *w*_*t*+1_ is the updated global model at round *t* + 1. *K* is the number of participating clients. *n*_*k*_ is the number of data samples held by client *k*. *N* = $\mathop \sum \nolimits_{k = 1}^{K} n_{k}$ is the total number of samples across all clients. $w_{t}^{k}$ is the local model update from client *k* at round *t*.

By integrating Differential Privacy with Federated Learning, the model ensures privacy preservation while maintaining robust performance.

#### Advantages and considerations in FL training

- *Data Privacy Protection*: Federated Learning ensures that only model updates are shared while sensitive patient information remains securely stored within each institution. This approach guarantees compliance with medical data privacy regulations.
- *Handling Data Heterogeneity*: In the medical sector, hospitals often have varying distributions of benign and malignant cancer cases. Some experiments employed non-IID data partitioning, demonstrating that the model could effectively handle data heterogeneity and outliers, validating its robustness in diverse scenarios.
- *Communication Efficiency*: FL optimizes communication bandwidth by transmitting only model updates instead of entire patient datasets. However, maintaining efficient communication among clients, the central server, and external sources is crucial for balancing model performance and network efficiency.
- *Scalability*: The FL framework is capable of scaling to more than ten clients while ensuring quality service across multiple healthcare institutions in a decentralized manner. However, inconsistencies in client participation or failures in communication may slow down the learning process.

## Evaluation metrics

The evaluation of the performance of machine learning models, especially those used in Federated Learning (FL) and Differential Privacy (DP), demands a precise set of performance metrics that can directly reflect classification accuracy, privacy leakage, and model convergence. Here we describe the mathematical details of some key metrics: accuracy, precision, recall, and F1-score, also giving the privacy leakage metric and the model convergence, both which are the paramount evaluation metrics for deciding over the efficiency of privacy-safeguarding models in federated learning.

### Measures for classification with model performance

Binary classification of breast cancer tumors is the primary task in this study. The performance of classification was measured by accuracy, precision, recall, and F1-score as these are the four compulsory evaluation criteria. These performance measures are derived from the confusion matrix grouped into four categories by the model predictions:

- *True Positives (TP)*: Malignant tumors correctly classified as malignant
- *True Negatives (TN)*: Benign tumors correctly classified as benign
- *False Positives (FP)*: Benign tumors incorrectly classified as malignant
- *False Negatives (FN)*: Malignant tumors incorrectly classified as benign

With these, the different evaluation metrics are derived considering either type of error, similar to really counting both FP and FN, which can be particularly important in medical applications^22,110^.

#### Accuracy

Accuracy is the most simplified metric and refers to the proportion of correctly classified cases to the total predictions. Mathematically, it is calculated as:

$${\text{Accuracy}} = \frac{TP + TN}{{TP + TN + FP + FN}}$$

where *TP* (True Positives) are the correctly classified positive cases. *TN* (True Negatives) are the correctly classified negative cases. *FP* (False Positives) are the incorrectly classified negative cases as positive. *FN* (False Negatives) are the incorrectly classified positive cases as negative.

While accuracy serves as a broad indicator of a model’s performance, it may not be reliable for highly imbalanced datasets where one class significantly outnumbers the other. In such cases, precision and recall are more informative metrics.

Precision, or Positive Predictive Value (PPV), is used to calculate the ratio between the correctly identified malignant cases and all cases that were predicted as malignant. It determines how many of the actual positive cases the model correctly identified, thereby minimizing the number of false-positive cases. This is particularly important in medical diagnostics, as reducing false positives helps avoid unnecessary treatments.

#### Precision

$${\text{Precision}} = \frac{TP}{{TP + FP}}$$

where *TP* (True Positives) are the correctly classified malignant cases. *FP* (False Positives) are the cases incorrectly classified as malignant.

A high precision value indicates that the model correctly identifies malignant tumors with minimal false positives, which helps reduce unnecessary medical interventions and patient discomfort.

#### Recall (Sensitivity)

Recall, also referred to as Sensitivity or True Positive Rate (TPR), evaluates the model’s ability to correctly identify malignant cases. It aims to minimize the number of false negatives, ensuring that malignant tumors do not go undetected. This is particularly crucial for early detection and treatment planning in cancer diagnosis.

$${\text{Recall}} = \frac{TP}{{TP + FN}}$$

where *TP* (True Positives) are the correctly classified malignant cases. *FN* (False Negatives) are the actual malignant cases incorrectly classified as benign.

A high recall value indicates that almost all malignant cases are detected, reducing the chances of misdiagnosis and ensuring that at-risk patients receive timely medical attention.

#### F1-score

The F1-score is the harmonic mean of precision and recall, providing a balanced evaluation metric, especially when the dataset is imbalanced. It effectively penalizes cases where either precision or recall is disproportionately high or low, ensuring a comprehensive measure of classification performance.

$${\text{F}}1{\text{ - score}} = 2 \times \frac{{{\text{Precision}} \times {\text{Recall}}}}{{{\text{Precision}} + {\text{Recall}}}}$$

where Precision measures the proportion of correctly predicted malignant cases among all predicted malignant cases. Recall (Sensitivity) evaluates how well the model identifies actual malignant cases.

By combining both precision and recall, the F1-score is particularly useful in healthcare applications, where minimizing both false positives and false negatives is critical to ensuring a reliable diagnostic model^111,112^.

#### Privacy budget (ε, δ)

When incorporating Differential Privacy (DP) into Federated Learning (FL), it is essential to quantify the level of privacy preserved during model training. This is done using the privacy loss metric, which is governed by the privacy budget parameters (*ε, δ)*^113^.

Differential privacy ensures that individual client data remains indistinguishable by introducing carefully calibrated Gaussian noise into model updates before they are aggregated. The privacy budget (*ε, δ)* determines the level of privacy protection:

- Epsilon (*ε*): Represents the extent to which information about an individual’s data can be inferred from the model’s output.
- A smaller *ε* provides stronger privacy but may reduce model utility by increasing noise.
- Delta (*δ)*: Defines the probability of privacy loss, ensuring that even in rare cases, an individual’s data cannot be distinguished.

The mathematical representation of a differentially private gradient update, as already discussed, is given by:

$$\tilde{g} = g + N\left( {0,\sigma^{2} } \right)$$

where $\tilde{g}$ is the differentially private gradient update. *g* is the original gradient computed by the client model. *N*(0, *σ*^2^) is Gaussian noise with variance *σ*^2^, ensuring privacy preservation. By varying *ε* and *δ*, the privacy-accuracy trade-off can be assessed. A lower *ε* enforces stronger privacy but may degrade model accuracy due to excessive noise. This trade-off is critical in medical applications, where both data privacy and model reliability are paramount.

## Results and discussion

This section discusses the detailed performance analysis of the proposed FL model with DP for breast cancer diagnosis against a traditional centralized machine learning model. It also explicitly underlines the discussion on the effectiveness of FL in preserving data privacy while offering good predictive performances. Furthermore, the section provides insight into the privacy-accuracy trade-offs, comparative analysis with alternative techniques for ensuring data privacy, and the applicability of the same in practical healthcare scenarios.

### Model performance comparison

**Fig. 14:**
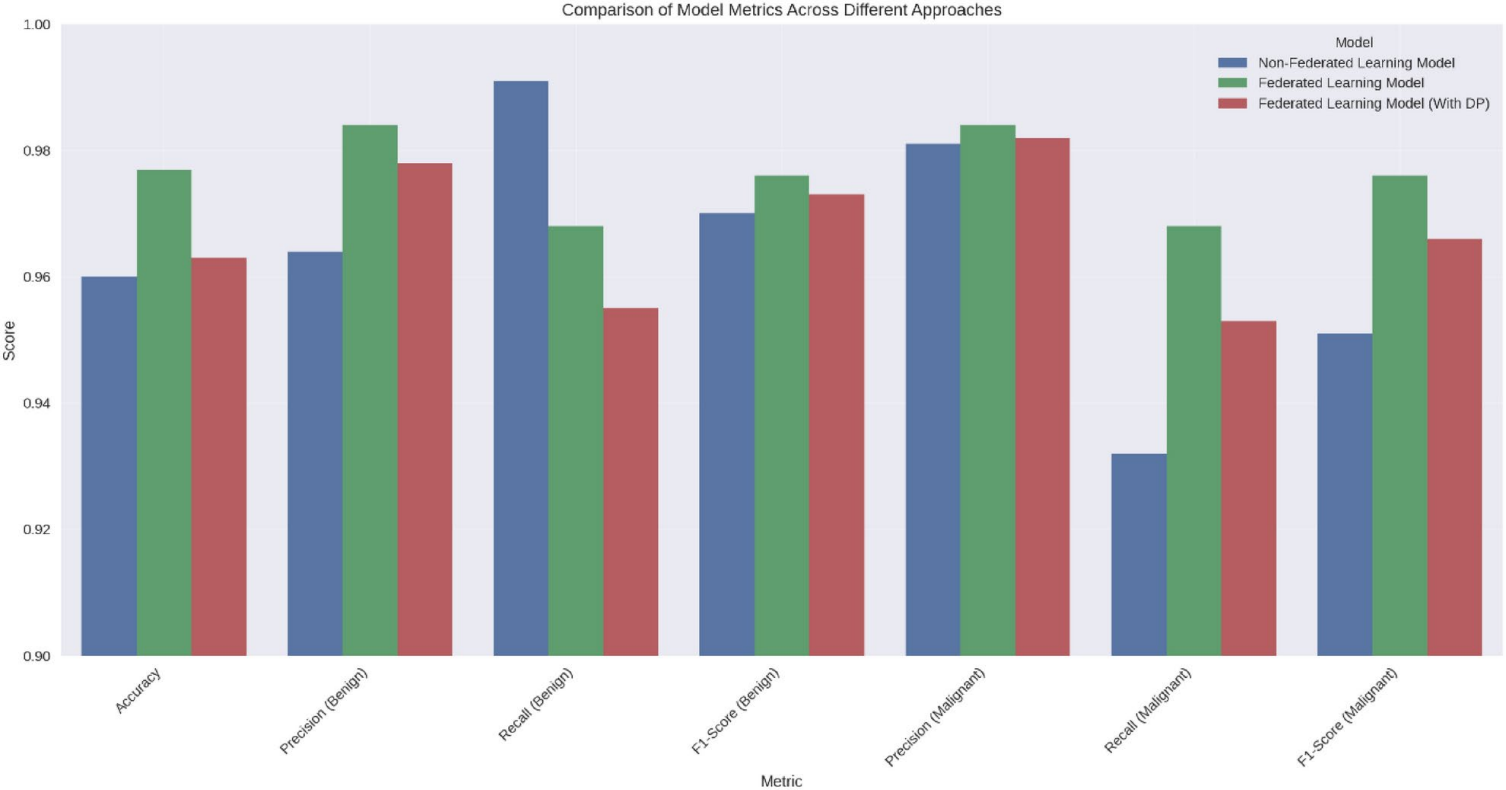
Visual comparison of performance metrics for Non-Federated Learning, Federated Learning, and Federated Learning with Differential Privacy.

The primary objective of this evaluation is to assess the performance of Federated Learning (FL) with and without Differential Privacy (DP) in comparison to a conventional non-FL machine learning approach. All models’ performances are evaluated based on the key performance metrics of accuracy, precision, recall, and F1-score to identify whether the introduction of FL and DP diminishes model performance in its predictive capabilities. Table 9 highlights the comparative performance of FL, FL-DP, and non-FL models in terms of accuracy, precision, recall, and F1-score. This table illustrates how integrating DP into FL impacts model performance while ensuring stronger privacy preservation. As shown in Table 9, it can be observed that FL yields an accuracy of 0.977, outperforming the non-FL model, which has an accuracy of 0.960, hence cementing the advantage of learning in a decentralized manner. The FL model trained with DP indeed reports a slight drop in accuracy but still manages to attain a better accuracy than the non-FL model, which is expected over the result of introduced noise for privacy preservation. Precision and recall are relatively similar across all models, indicating that FL retains predictive capability under privacy constraints. Figure 14 shows the visual comparison of model performance metrics across different learning approaches.

**Table 9:** Comparison of evaluation metrics across different models.

Metric	Non-FL model	FL model	FL model with DP
Accuracy	0.960	0.977	0.961
Precision (Benign)	0.964	0.984	0.978
Recall (Benign)	0.991	0.968	0.961
F1-Score (Benign)	0.970	0.976	0.974
Precision (Malignant)	0.981	0.984	0.983
Recall (Malignant)	0.932	0.968	0.959
F1-score (Malignant)	0.951	0.976	0.966

Although Table 9 compares different models, it is important to consider the impact of dataset heterogeneity. FL performs better than non-FL since it can learn from decentralized data even under non-IID conditions. On the addition of Differential Privacy, accuracy reduces from 0.977 to 0.961 because of the introduction of noise but such a trade-off cannot be avoided when maintaining privacy but is tolerable in the context of clinical use. FL also enables improved generalizability across institutions, significant in real-world applications in healthcare environments where the data would be heterogeneous.

**Fig. 15:**
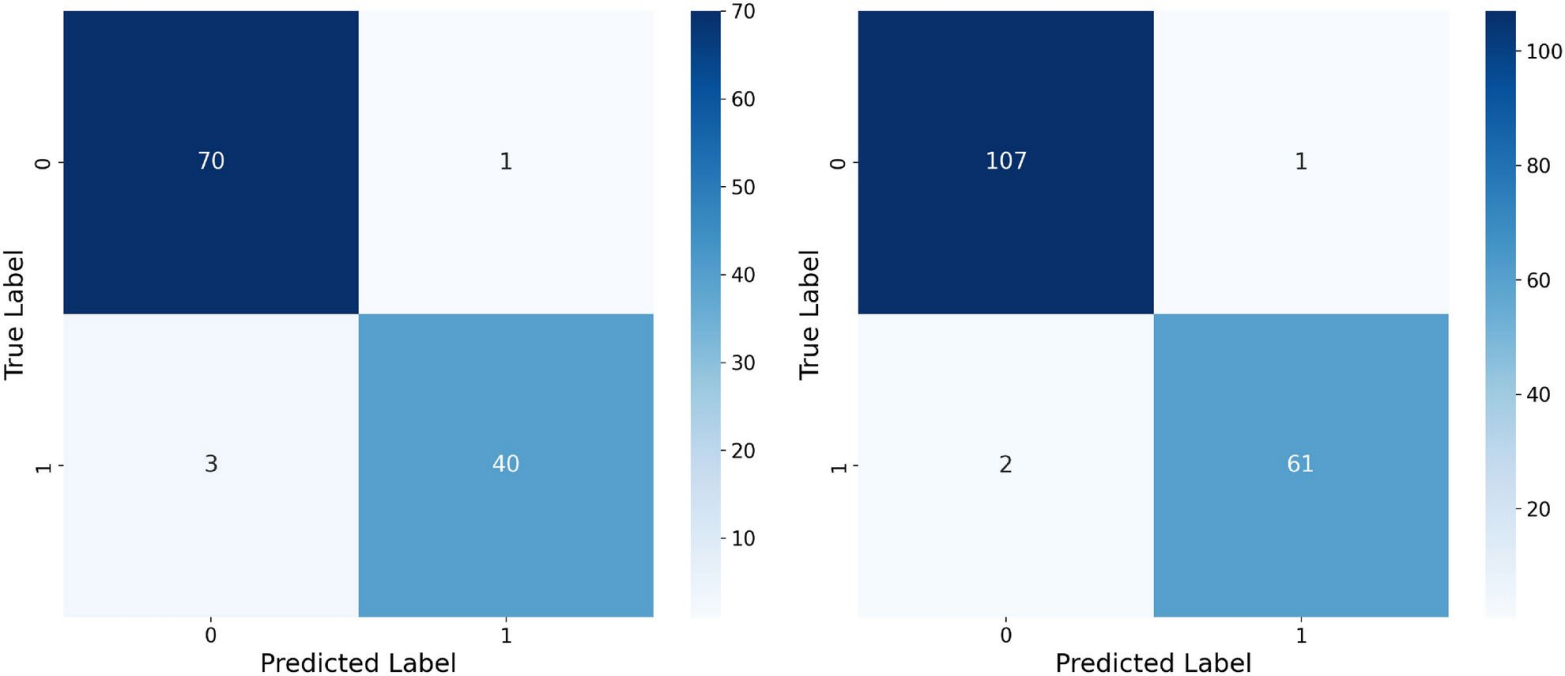
Confusion matrices of the non-federated and federated learning models.

A more detailed examination of the confusion matrices as shown in Fig. 15 brings a greater insight into the benefits of FL over traditional non-FL methods, at least in terms of minimizing misclassifications in both benign and malignant cases. From a clinical point of view, both false positives (i.e., wrong classification of a benign tumor as malignant) and false negatives (i.e., wrong diagnosis of malignant tumor as benign) need to be minimized to guarantee correctness and timely diagnosis.

These findings show that the FL model significantly reduces misclassifications compared to the non-FL model, reflecting its superior capability of generalizing decentralized datasets. With increased discrimination power, FL performs well in identifying various kinds of patterns from distributed data sources, reflecting model robustness and diagnostic precision. Notice that Federated Learning does not sacrifice predictive performance but instead enhances it. By the nature of the decentralization in FL, several institutions can collaboratively train a global model while keeping local patient data, thus guaranteeing data privacy while taking advantage of heterogeneous medical datasets. Within this collaborative learning approach, the feature extraction will be stronger, and the decision-making will result in greater classification accuracy along with improved clinical applicability.

**Fig. 16:**
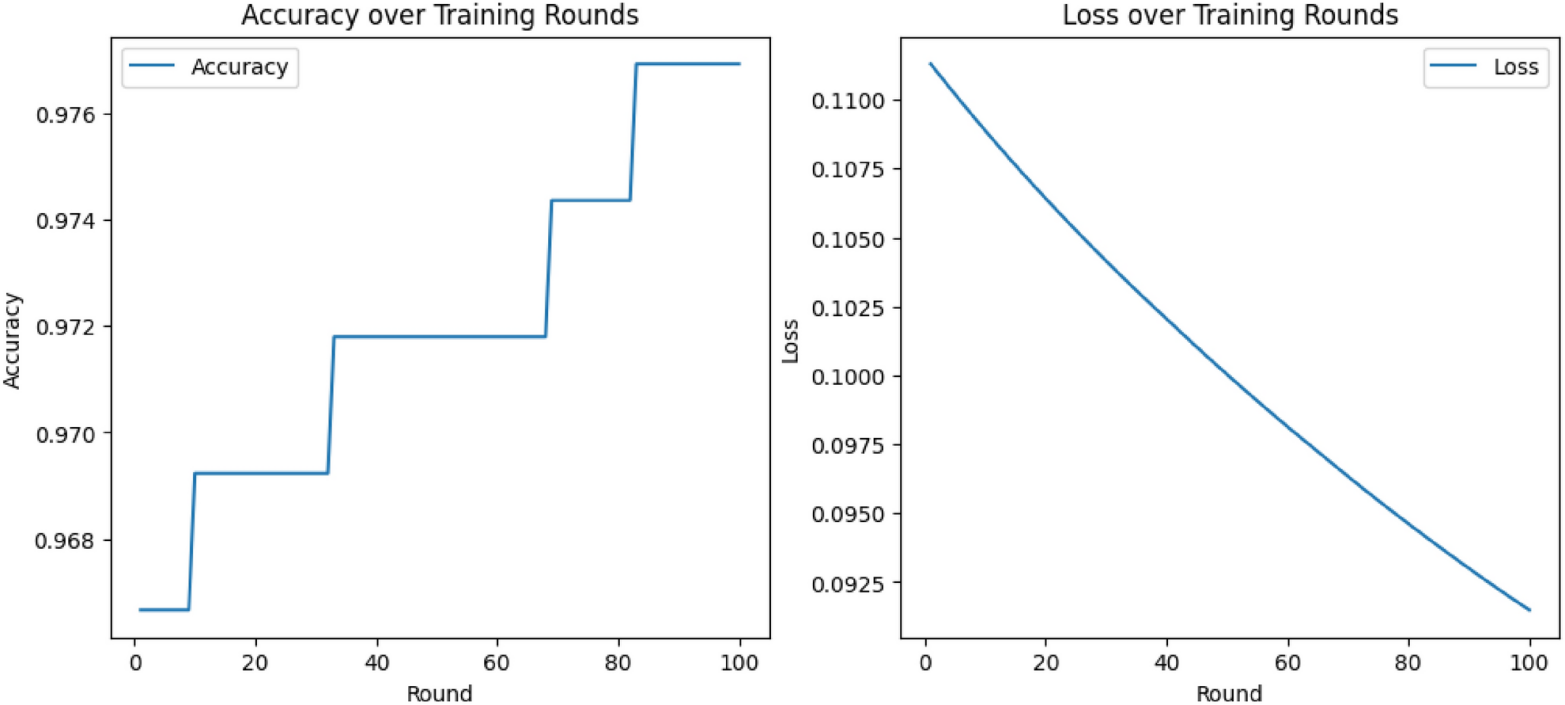
Accuracy and loss over training rounds.

Figure 16 shows the training curve of the FL model-developing precision and loss over several training rounds. Conversely, as training continues, one can observe that the model’s precision constantly increases, confirming the fact that it learns to distinguish between benign and malignant cases. At the same time, the loss function constantly decreases, which means that a model can minimize its classification errors round by round. It also sets a trend for the stability and performance of FL on medical classification tasks.

**Fig. 17:**
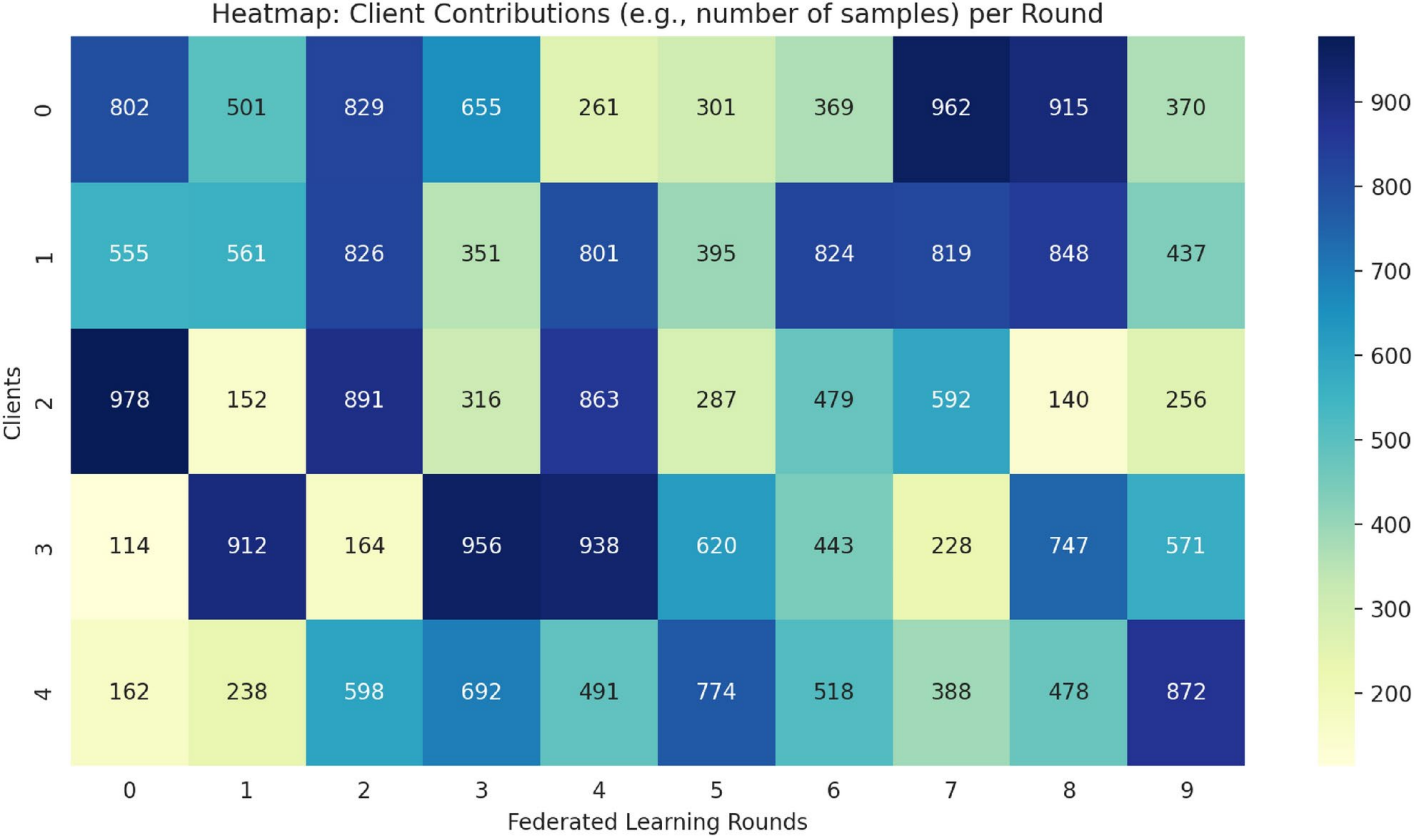
Heatmap- Client contributions per round.

Figure 17 demonstrates the heterogeneity of client contribution in training iterations. Client participation in federated learning can be heterogeneous due to differences in data quantities, computing resources, and local model updates. The heatmap highlights how some contribute more updates, while others contribute fewer updates. It is extremely crucial to understand these differences in terms of optimizing aggregation procedures and making sure each institution contributes proportionally to the global model.

**Fig. 18:**
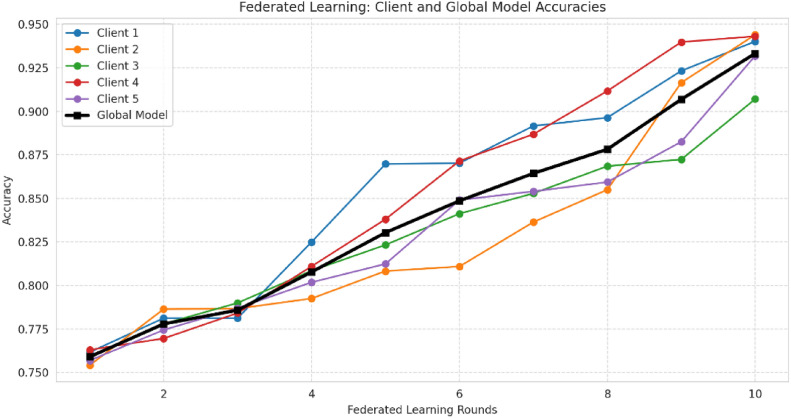
Federated learning: Client and Global model accuracies.

Figure 18 depicts the trend of accuracy of each client model and the global FL model. From the graph, each of the local client models, after several rounds of iterative training, has achieved some accuracy and converged to a well-optimized global model. It is obvious that FL enabled heterogeneous knowledge sharing across the clients without sharing data directly with each other while maintaining high-performance consistency.

The FL model achieves robust predictive performance across multiple rounds of training, further indicating that FL-based learning can be leveraged to achieve comparable performance to the traditional centralized model without patient privacy compromise. These findings further confirm that Federated Learning is indeed a comprehensive and effective solution for medical classification tasks because, besides preserving data security and privacy, it offers high classification accuracy. Therefore, the reduced misclassification rate, the stable learning curve, and the good balance in contribution from federated clients make FL highly promising for real-world healthcare applications where the security and privacy of data preservation, together with diagnostic accuracy, are of utmost importance.

### Privacy vs. accuracy trade-off

**Fig. 19:**
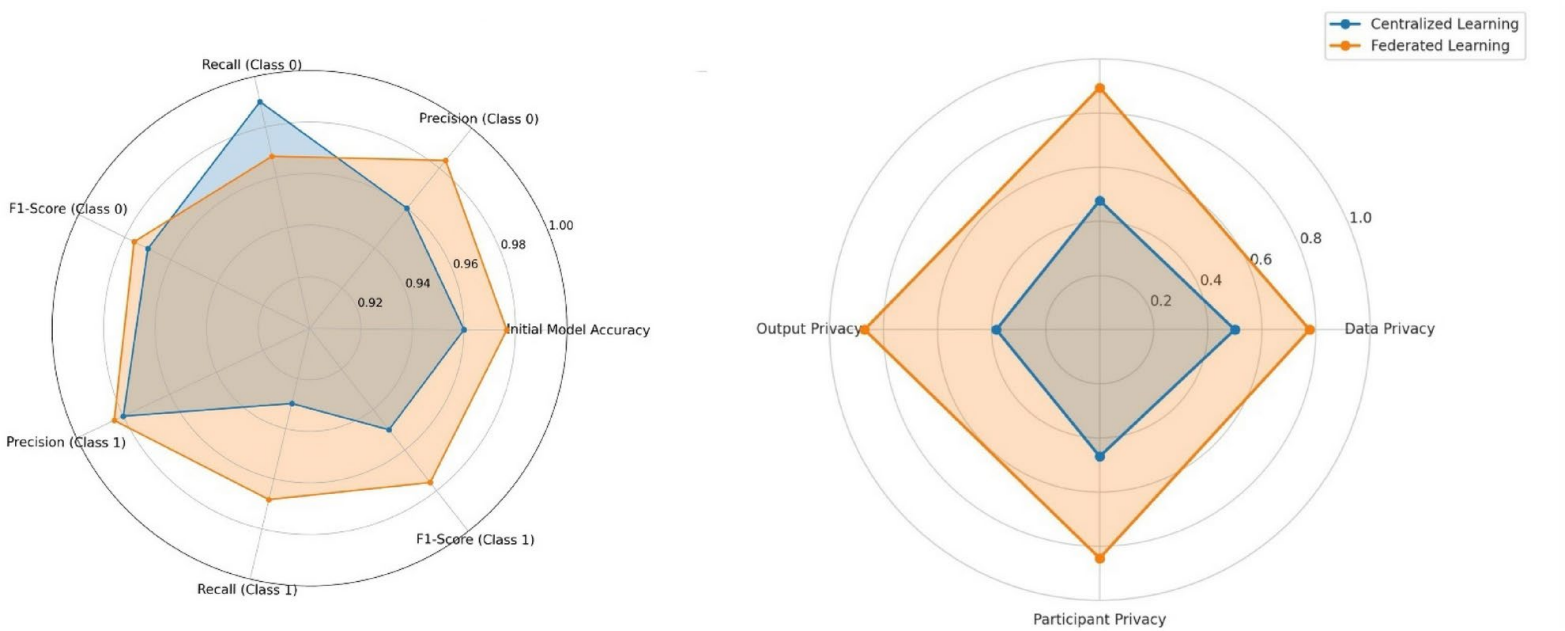
Comparison of evaluation metrics (left) and privacy preservation (right) for centralized vs. federated learning.

Another important characteristic of federated learning is that it maintains privacy. federated learning accepts the training distributed across multiple institutions, whereas normal machine learning models would demand the centralization of data. It is this non-requirement of centralization of data that builds privacy preservation. This keeps the patient data from spreading out into external institutions and confines it to a local field. Figure 19 presents a side-by-side comparison of evaluation metrics and privacy preservation, demonstrating the trade-offs between model performance and data security in federated learning. Privacy preservation plays a significant role in healthcare as confidentiality about the patient is kept at the highest priority. This federating learning model has maintained the privacy factor without allowing the degradation of the performance of the model.

**Fig. 20:**
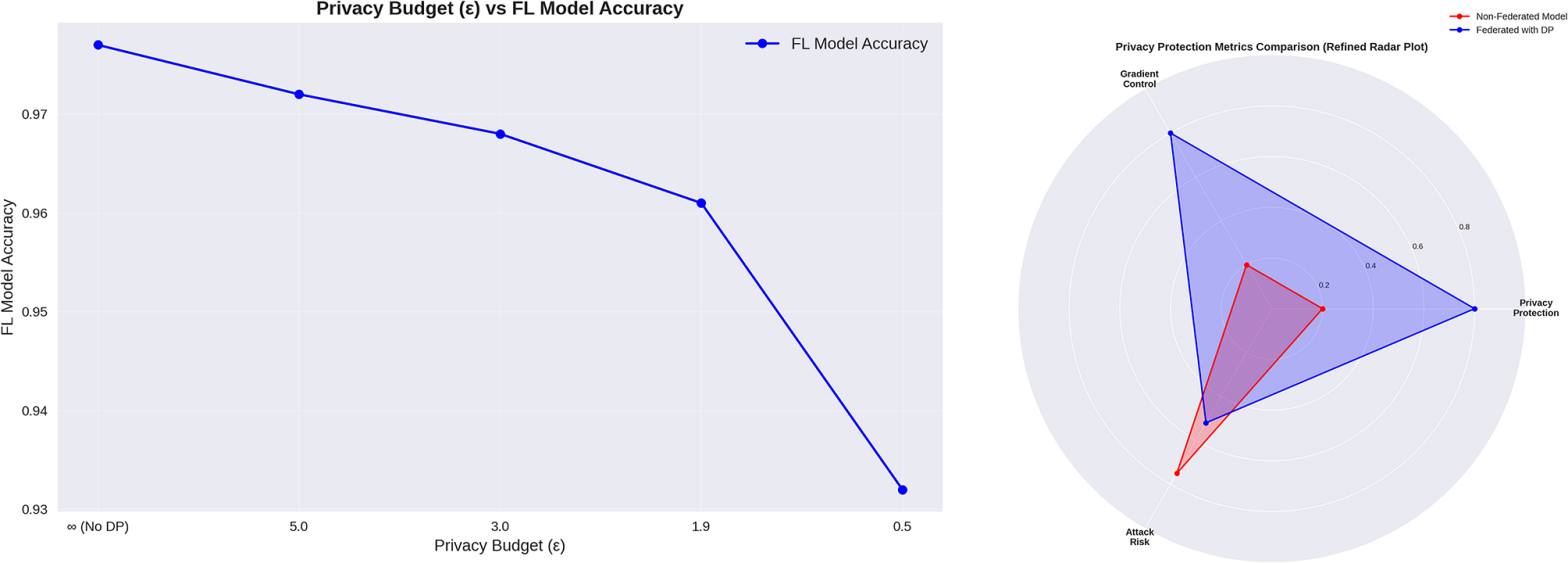
Impact of privacy budget (ε) on FL model accuracy (left) and comparative privacy protection analysis of FL-DP vs. non-federated models (right).

While FL provides substantial privacy advantages, incorporating Differential Privacy (DP) introduces a fundamental trade-off between privacy protection and model accuracy. The privacy budget *ε* determines the level of privacy preservation, with low *ε* values usually implying strong privacy at the cost of higher accuracy degradation. Table 10 summarizes the impact of different privacy budgets on the accuracy of the FL model. It can be inferred from this table that a higher privacy setting results in an accuracy drop, while an optimum trade-off at *ε* = 1.9 keeps the accuracy high. Table 11 presents a comparison of privacy protection metrics between Non-Federated and Federated Learning models. Figure 20 shows that the visual representation of how different *ε* values affect accuracy: an optimal privacy budget, *ε* = 1.9, would be chosen since it guarantees adequate privacy without significant deterioration in performance. This suggests that a good balance between privacy and utility in privacy-preserving FL systems is vital, especially in healthcare applications where high diagnostic accuracy must be maintained.

**Table 10:** Impact of privacy budget (*ε*) on federated learning model accuracy.

Privacy budget (*ε*)	FL model accuracy
∞ (No DP)	0.977
5.0	0.972
3.0	0.968
1.9 (Chosen DP settings)	0.961
0.5 (Strongest DP)	0.932

**Table 11:** Comparison of privacy protection metrics between non-federated and federated learning models.

Metric	Non-federated learning model	Federated learning model (With DP)
Privacy Budget (*ε*)	No DP (Unprotected data exposure)	1.9 (Good privacy protection)
Gradient Sensitivity (L2 Norm)	No Clipping (Unrestricted gradients)	1.8 (Gradient clipping applied)
Membership inference attack risk	0.75 (High privacy risk)	0.52 (Better privacy)

The radar plot in Fig. 20 has provided a visual comparison of the privacy-related performance of two models-Non- Federated Learning Model and the Federated Learning Model with Differential Privacy. The plot has considered these models based on three important privacy-preserving dimensions:

- *Privacy Protection*: It refers to the extent a model is capable of guarding sensitive data against possible leakage. In comparison, the FL with DP has much better privacy protection than the non-FL model. This is because FL does not share data directly among clients, and in addition, DP defends by adding noise to prevent the reconstruction of data.
- *Gradient Control*: Gradient control characterizes the model’s ability to guard gradient information during training so it might prevent adversarial attacks or model inversion. In this case, the FL with DP model allows for greater gradient control, aptly reflected in the higher coverage of the blue polygon. The non-FL model has greater leakage in the gradient and thus is more susceptible to these kinds of privacy attacks, such as model inversion or membership inference attacks.
- *Attack risk*: This indicates the risk of the model being attacked regarding privacy, such as by membership inference attacks or model inversion attacks. Along this axis, more coverage of the red non-FL model means a greater risk of an attack. The FL with DP model strongly reduces the risk of an attack; thus, it is much safer for use in privacy-sensitive areas like health care and medical diagnosis.

Although DP ensures strong privacy protection, it is computationally costly because of the addition of noise in model updates. Our experiments show that larger privacy budgets (ε) result in minor accuracy drops but also increase training time because of the added noise perturbation operations. With the considered privacy budget (ε = 1.9), per-round training time was around 25% longer than in the non-DP FL model. This emphasizes the importance of efficient privacy-aware training methods that save computational costs while ensuring privacy protection, particularly in resource-scarce healthcare settings.

#### Comparison with other privacy-preserving techniques

**Fig. 21:**
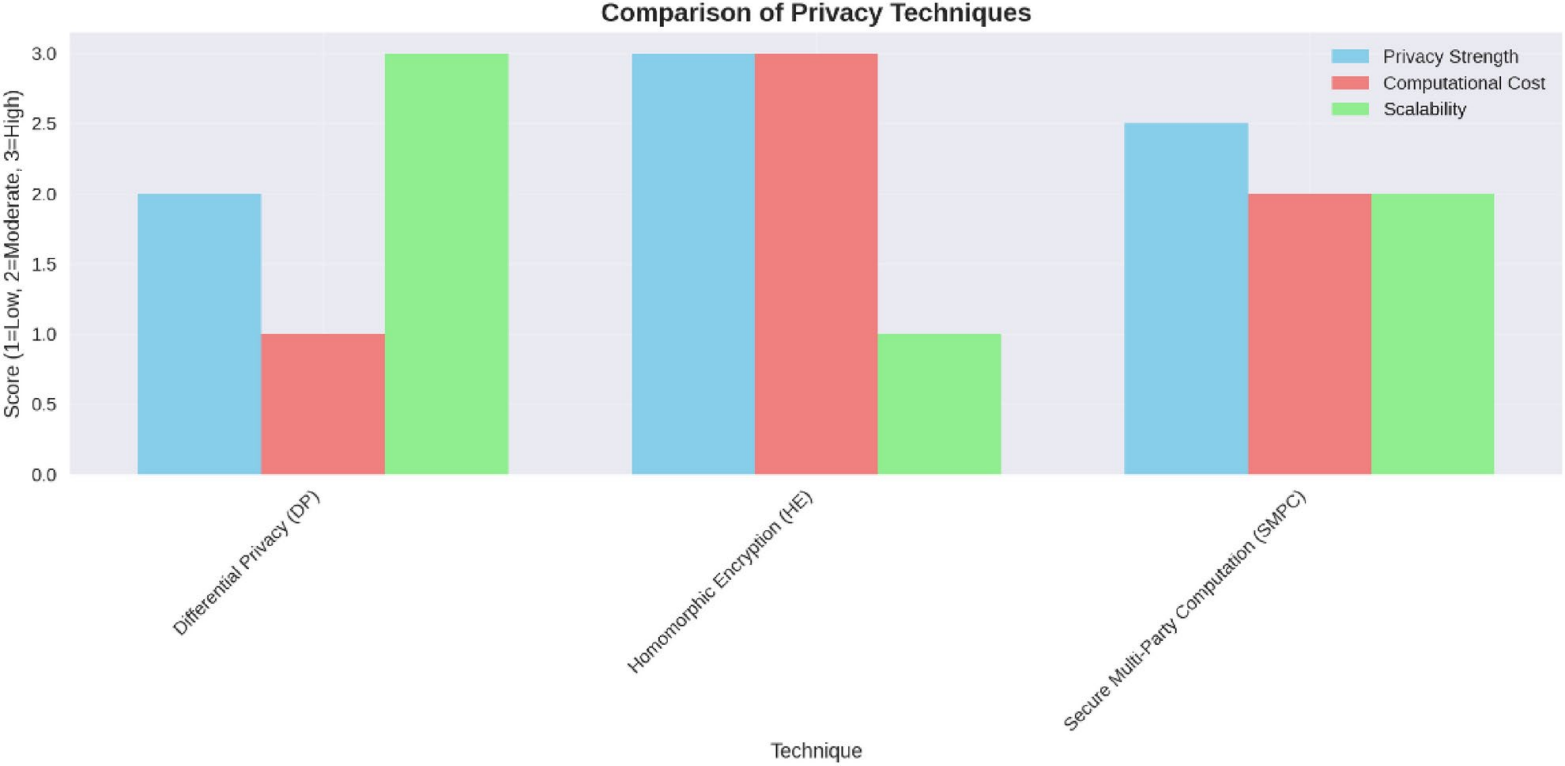
Comparison of privacy techniques.

For privacy preservation in federated healthcare applications, multiple techniques are used, such as Differential Privacy, Homomorphic Encryption, and Secure Multi-Party Computation. In this regard, a comparison of these techniques according to the strength of privacy, computational cost, scalability, and feasibility for integration with FL has been presented in Table 12 and Fig. 21. From the results, one can observe that DP strikes a good balance in privacy and efficiency, hence it is the most practical choice for FL in healthcare.

**Table 12:** Comparison of privacy techniques based on privacy strength, computational cost, and scalability.

Technique	Privacy strength	Computational cost	Scalability
Differential privacy (DP)	2 (Moderate)	1 (Low)	3 (High)
Homomorphic encryption (HE)	3 (High)	3 (High)	1 (Low)
Secure multi-party computation (SMPC)	2.5 (Moderate-high)	2 (Moderate)	2 (Moderate)

Unlike HE and SMPC, which impose high computational overheads, DP is lightweight and scalable across multiple hospital networks. HE provides stronger privacy guarantees but significantly impacts training speed and efficiency, making it impractical for real-time medical applications. Similarly, SMPC, while offering high privacy protection, faces scalability limitations due to its reliance on complex cryptographic operations. Figure 16 illustrates the comparative strengths and weaknesses of these techniques, highlighting why DP is the preferred choice for FL in real-world healthcare settings.

#### Patient-centric benefits of FL-DP in breast cancer diagnosis

The application of Federated Learning with Differential Privacy (FL-DP) in breast cancer diagnosis not only enhances data security but also significantly improves patient-centered outcomes. By leveraging a decentralized model learning from diverse hospital datasets, FL-DP accelerates AI-assisted diagnosis, reducing the time required for second opinions and facilitating timely treatment decisions. Moreover, FL-DP promotes clinicians’ and patients’ trust and transparency, with the promise of keeping sensitive medical information within the hospital walls, countering typical concerns regarding the secrecy of data in AI-supported health solutions. Enhanced generalizability of FL-DP models on heterogeneous patient populations also translates into more accurate and fair diagnostic estimates, precluding potential training bias-related risks from central data. Lastly, FL-DP supports personalized treatment plans with the ability to enable AI models to learn across institutions without violating confidentiality. This ensures personalized treatment suggestions may be informed with a wider set of patient data while maintaining high levels of privacy preservation. Such benefits make FL-DP a best-fit technology for large-scale AI deployment in healthcare, with a primary balance between privacy guarantee and high-accuracy diagnostic efficiency.

#### Real-world applicability and challenges

Despite all the benefits, there are various challenges in deploying FL with DP in real-world healthcare applications. First, scalability is an important problem since FL requires high-bandwidth communication among multiple hospitals. Thus, efficient aggregation strategies like FedProx and Adaptive FedAvg should be employed. Second, DP will bring extra computation overhead since adding noise needs more computational resources. This could be a bottleneck for those low-resource hospitals without advanced computational infrastructures.

A critical risk in FL involves malicious clients seeking to inject adversarial data or manipulate model updates. Although FL considers all participating clients honest, adversarial attacks can seriously affect model performance. With that, concentration must be directed toward Byzantine-Resistant FL techniques and Secure Aggregation for increased robustness against possible threats. Similarly, FL in healthcare will have to be designed with strict consideration for patient privacy, guided by HIPAA and GDPR legislation, imposing legal limitations on data sharing and processing. Ensuring regulatory compliance while sustaining high performance remains an ongoing challenge.

Despite these advantages, the deployment of FL-DP in healthcare must address key ethical and regulatory concerns. Unlike traditional machine learning, FL operates without centralizing patient data. However, regulatory frameworks such as HIPAA and GDPR mandate that patient data privacy be maintained, necessitating transparent patient consent mechanisms. Ensuring that patients understand how their data contributes to federated models while maintaining individual privacy remains a significant challenge. Similar trials in pathology and radiology are ongoing, signaling FL’s feasibility for full deployment in the clinic. Another challenge involves obtaining informed patient consent for federated learning. Unlike centralized AI models, FL requires hospitals to communicate model updates rather than raw data. Institutions must develop clear consent policies and explain FL benefits to patients, ensuring ethical adherence.

It would be interesting for further studies to include the integration of blockchain technology with FL and explore the additional layers of security and auditability in a federated training environment.

#### Proposed clinical workflow for FL-DP integration in breast cancer diagnosis

The application of Federated Learning with Differential Privacy (FL-DP) to breast cancer diagnosis adopts a systematic clinical workflow for simplicity of deployment in real-world healthcare environments. The proposed workflow is:

1. *Data Collection and Local Processing*: Patient imaging data (e.g., mammograms, histopathology slides) are retained by hospitals and diagnostic centers locally. Each institution trains a local model on its own private data set to satisfy requirements like HIPAA and GDPR.
2. *Federated Model Training*: Instead of sharing sensitive patient records, hospitals collaborate by sharing encrypted model updates to a central aggregation server. This allows for real-time adaptation to evolving patient information without data centralization.
3. *Diagnosis Support & Decision Assistance*: FL-DP, the global model, provides oncologists with AI-driven diagnostic support in the form of tumor classification (benign or malignant) data, cancer progression prediction, and personalized treatment recommendations.
4. *Model Updates & Continuous Learning*: With fresh available patient information, the FL-DP model is continuously retrained to yield better accuracy without violating patient privacy. Continuous learning enhances diagnostic accuracy and responsiveness in hospitals. Under using this workflow, FL-DP is consistent with current clinical decision-making workflows, and thus deployable in real oncology departments.

For instance, FL-DP can be utilized to facilitate AI-assisted mammography screening in which hospitals exchange their data to be used for a common model. This will assist oncologists in making improved predictions, which will improve early breast cancer detection while patient data remains within the hospital.

## Limitations and future work

While Federated Learning with Differential Privacy (FL-DP) offers a robust framework for privacy-preserving AI in healthcare, it faces several limitations that must be addressed for widespread adoption.

One of the primary challenges is the trade-off between privacy and model accuracy. Slight degradation in accuracy of the models due to the addition of noise in DP, which, though necessary for privacy, reduces the predictive power; this may be tamed by careful fine-tuning of the privacy budget *ε* and *δ.* Another significant limitation is the high computational cost associated with FL-DP. Unlike centralized learning, which relies on a single, powerful server, federated learning distributes computations across multiple institutions, necessitating substantial hardware and processing power. This makes FL-DP less feasible for resource-constrained hospitals, where access to high-performance computing infrastructure is limited. Future work should explore lightweight FL architectures and more efficient aggregation techniques to enable broader adoption.

Data heterogeneity is yet another key issue in federated healthcare AI. Medical data within hospitals tends to have non-independent and identically distributed (non-IID) features, which results in inconsistent global model convergence. This difference in data distributions may introduce bias in AI predictions and necessitate the creation of more sophisticated aggregation techniques than mere averaging methods to enhance generalizability.

Additionally, the study assumes that the dataset used is balanced and preprocessed, whereas real-world clinical data often suffers from class imbalances and missing records. In a practical hospital setting, malignant cases would normally be fewer in number compared to benign ones and thus affect model performance. To solve this, FL models must incorporate adaptive weighting to cope with imbalanced classes and build strong imputation methods to deal with missing attributes. Future research should focus on evaluating FL models in real-world hospital datasets to validate their effectiveness under practical clinical conditions.

From a deployment point of view, there are various challenges to the widespread application of FL-DP. One of the challenges is that there is no standardization of FL protocols among healthcare organizations. Hospitals have varying IT infrastructures and regulatory environments, which create challenges in adopting a standardized FL system. There needs to be an interface between medical AI researchers, healthcare professionals, and regulators to come up with normalized FL implementation standards that guarantee interoperability and conformity across organizations.

Another significant challenge is Electronic Health Record (EHR) interoperability. FL-DP integration with current hospital systems necessitates seamless compatibility so as not to disrupt clinical workflows. Creating FL-compatible APIs that enable seamless integration with EHR systems is vital for the practical implementation of FL-DP in hospitals.

Outside these technical constraints, ethical and legal issues also need to be dealt with. Patient consent and data governance policies within FL are a challenge since the participants might not be fully conscious of how their data is being used for global model training. Having clear patient participation frameworks in place and meeting data protection laws like HIPAA and GDPR will be critical for trust building and regulatory approval.

To enhance the scalability and security of FL-DP, future studies can investigate adaptive DP mechanisms that adaptively update privacy budgets in real-time depending on data sensitivity, thus achieving minimal accuracy loss while ensuring robust privacy protection. Moreover, the combination of blockchain technology with FL can further increase security via secure model aggregation and decentralized trust management, making FL-DP more robust against adversarial attacks.

By addressing these challenges, future advancements in FL-DP can lead to the development of more secure, scalable, and privacy-friendly machine learning frameworks tailored for healthcare. These improvements will not only strengthen data security but also ensure high diagnostic accuracy, making AI-driven healthcare solutions both ethically sound and clinically reliable.

## Conclusion

The study demonstrates that Federated Learning (FL) with Differential Privacy (DP) effectively balances data privacy and model accuracy for breast cancer diagnosis. The results show that FL outperforms traditional centralized models, proving its ability to generalize across decentralized data without compromising predictive performance. Despite FL with DP involving slight accuracy concessions, it maintains strong diagnostic performance while yielding enhanced privacy protection. Confusion matrix analysis proves that FL is effective in reducing misclassification rates, a dimension that is critical to minimizing false positives and false negatives in healthcare. Privacy-accuracy trade-off analysis shows that the use of an effective privacy budget is essential in maintaining privacy while maintaining high levels of accuracy. In addition, comparative analysis of DP, Homomorphic Encryption (HE), and Secure Multi-Party Computation (SMPC) points to DP as the most viable method owing to its minimal computational overhead and scalability in real-world healthcare settings. Despite its advantages, scalability, computational overhead, and issues of regulatory compliance remain. Future studies should resolve issues of adaptive DP methods, blockchain-based model aggregation for secure model aggregation, and experiments on large-scale medical datasets to further enhance privacy-preserving artificial intelligence in healthcare. FL with DP acts as a bridge between privacy and performance, aiding in the development of secure and scalable AI-driven medical solutions.

## Data Availability

The Breast Cancer Wisconsin Diagnostic dataset used in this research is publicly accessible at the UCI Machine Learning Repository: Breast Cancer Wisconsin (Diagnostic)—UCI Machine Learning Repository https://archive.ics.uci.edu/dataset/17/breast+cancer+wisconsin+diagnostic.
